# Attenuating metal-substrate conjugation in atomically dispersed nickel catalysts for electroreduction of CO_2_ to CO

**DOI:** 10.1038/s41467-022-33692-0

**Published:** 2022-10-14

**Authors:** Qiyou Wang, Kang Liu, Kangman Hu, Chao Cai, Huangjingwei Li, Hongmei Li, Matias Herran, Ying-Rui Lu, Ting-Shan Chan, Chao Ma, Junwei Fu, Shiguo Zhang, Ying Liang, Emiliano Cortés, Min Liu

**Affiliations:** 1 Hunan Joint International Research Center for Carbon Dioxide Resource Utilization, State Key Laboratory of Powder Metallurgy, School of Physics and Electronics, Central South University, Changsha, 410083 China; 2 Nanoinstitut München, Fakultät für Physik, Ludwig-Maximilians-Universität München, 80539 München, Germany; 3 National Synchrotron Radiation Research Center, 300 Hsinchu, Taiwan; 4 College of Materials Science and Engineering, Hunan University, Changsha, 410082 China; 5 College of Food Science and Engineering, Central South University of Forestry and Technology, Changsha, 410004 China

**Keywords:** Energy, Electrocatalysis, Electrocatalysis, Heterogeneous catalysis

## Abstract

Atomically dispersed transition metals on carbon-based aromatic substrates are an emerging class of electrocatalysts for the electroreduction of CO_2_. However, electron delocalization of the metal site with the carbon support via d-π conjugation strongly hinders CO_2_ activation at the active metal centers. Herein, we introduce a strategy to attenuate the d-π conjugation at single Ni atomic sites by functionalizing the support with cyano moieties. In situ attenuated total reflection infrared spectroscopy and theoretical calculations demonstrate that this strategy increases the electron density around the metal centers and facilitates CO_2_ activation. As a result, for the electroreduction of CO_2_ to CO in aqueous KHCO_3_ electrolyte, the cyano-modified catalyst exhibits a turnover frequency of ~22,000 per hour at −1.178 V versus the reversible hydrogen electrode (RHE) and maintains a Faradaic efficiency (FE) above 90% even with a CO_2_ concentration of only 30% in an H-type cell. In a flow cell under pure CO_2_ at −0.93 V versus RHE the cyano-modified catalyst enables a current density of −300 mA/cm^2^ with a FE above 90%.

## Abstract (web summary)

Electroreduction of CO_2_ on single atom catalysts is often hindered by electron delocalization of the metal sites. To improve CO_2_ activation, here the authors functionalize the carbon support with cyano moieties, thereby attenuating metal-substrate conjugation and improving CO_2_ to CO conversion.

## Introduction

CO_2_ electroreduction reaction (CO_2_RR) is one of the most attractive strategies to restrain the greenhouse effect and meanwhile produce CO for Fischer-Tropsch synthesis^1–3^. Recently, *d*-block single atom catalysts (SACs) are considered to be among the most promising ones for CO production, due to their atomically dispersed characteristic, which endows SACs with definite active centers, stable coordination environment and maximum atom utilization^4^. In most cases, SACs are accomplished through the controlled deposition of metallic atoms on carbon-based substrates, such as carbon nitride (C_3_N_4_). Such substrates usually possess an inherent π(pi)-conjugated system, which provides remarkable electrical conductivity and stability to the entire electrocatalyst^5,6^. However, this type of carbon-based matrix also promotes a strong *d*-π conjugation between the *d* orbitals of the metal centers and the π orbitals of the substrate^7^. This undesired *d*-π conjugation facilitates electron transfer from the metal center towards the substrate, vastly hampering the activation of CO_2_ molecules on metal sites of SACs^8^. Therefore, the strong delocalization of electrons introduced by the support turns out to be detrimental for catalysis applications.

Many efforts have been devoted to regulate the electronic states of metal sites for CO_2_ activation through adjusting their coordination environment^9,10^. For example, Jiang et al. deliberately altered N coordination numbers to optimize the electronic states of Ni sites and the CO_2_ activation process, leading to a turnover frequency (TOF) of ~1622 h^−1^ (h^−1^)^11^. Zhuang et al. constructed N-bridged bimetallic Co and Ni sites to enrich their electron density and promote CO_2_ activation. As a result^12^, their TOF could reach to ~2049 h^−1^. To further enhance the electron density of Ni sites, Zhao et al. doped sulfur atoms into Ni SACs to substitute N coordination atoms. With the increase of electron density, the CO_2_ activation energy barrier was obviously decreased^13^, resulting in a notable TOF of ~3965 h^−1^. Unfortunately, the efficiency of CO_2_RR is still unsatisfactory for SACs, because CO_2_ activation energy barrier remains a high level on metal sites. On the other hand, inert carbon-based materials such as nitrogen doped carbon, graphitized C_3_N_4_ and graphdiyne feature abundant delocalized π orbits. The establishment of metal-substrate system in SACs inevitably leads to a strong *d*-π conjugation. Therefore, it is significant to properly attenuate *d*-π conjugation on metal sites to increase the electron density for CO_2_ activation while still ensuring the structural stability.

Herein, we attenuated the *d*-π conjugation in atomically dispersed Ni sites embedded in C_3_N_4_ through the introduction of cyano groups (−CN). Density functional theory (DFT) calculations indicate a favorable CO_2_ activation on the Ni@C_3_N_4_-CN catalyst due to the attenuated *d*-π conjugation. The predicted theoretical results were confirmed experimentally. Ni@C_3_N_4_-CN exhibits a prominent CO_2_RR performance with a TOF of ~22,000 hour^−1^ with a FE_CO_ ≥ 90% in an H-cell. Remarkably, the Ni@C_3_N_4_-CN still attains FE_CO_ ≥ 90% even at low CO_2_ concentrations. Additionally, the flow cell assembled with Ni@C_3_N_4_-CN reaches a current density of −300 mA/cm^2^ with a FE_CO_ ≥ 90%, meeting a desirable application prospect for industrialization. The superior CO_2_ activation on Ni@C_3_N_4_-CN compared to the intact C_3_N_4_ matrix was also confirmed by temperature program desorption (TPD) and electrocatalytic measurements. Furthermore, in situ spectroscopic analysis revealed that the formation of the *COOH intermediate is favored on the CN-modified SAC and this is one of the key steps to accelerate the CO_2_RR.

## Results and discussion

### Theoretical calculations

**Fig. 1:**
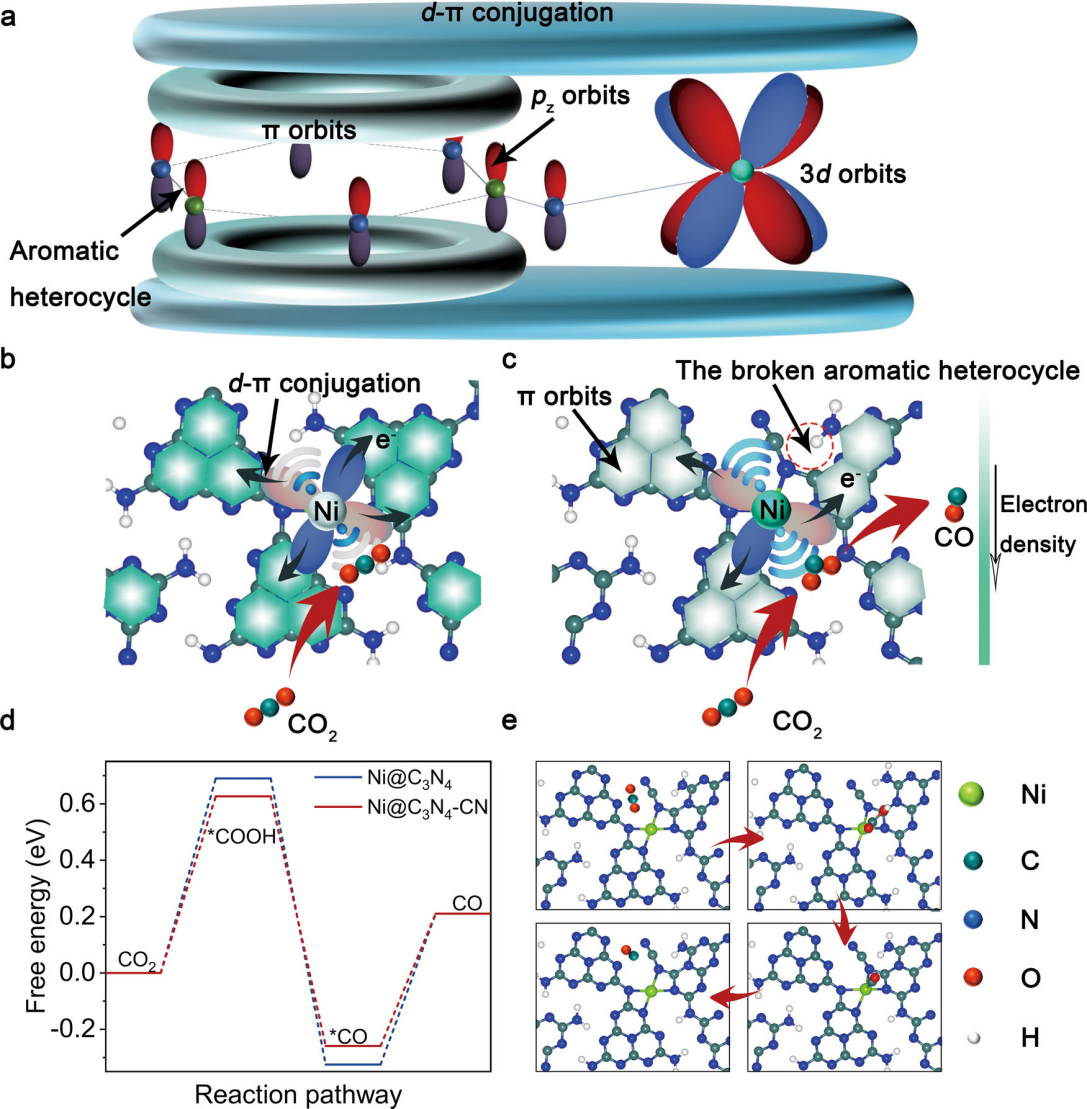
Theoretical calculations. **a** Schematic diagram for *d*-π conjugation. The effect of *d*-π conjugation on CO_2_ activation, **b** Ni@C_3_N_4_-CN and **c** Ni@C_3_N_4_. **d** Free energy diagram. **e** Structure and adsorption configurations of key intermediates on Ni@C_3_N_4_-CN.

To understand the effect of attenuated *d*-π conjugation for SACs, a theoretical analysis was initially performed on Ni@C_3_N_4_-CN. The C_3_N_4_ substrate containing π-conjugated aromatic heterocycles was selected as a substrate to load Ni sites with a general coordination number of 4 (Fig. 1 and Supplementary Fig. 1). Schematically, the electrons easily transfer from the Ni sites to the C_3_N_4_ substrate through a strong *d*-π conjugation^14^, leading to electronic delocalization and weaker CO_2_ activation ability at the Ni sites (Fig. 1b).

Conversely, our calculations predict a weaker *d*-π conjugation between the Ni sites and the carbon-based matrix for the C_3_N_4_-CN moieties. The poorer interaction between the metal and the support is a consequence of the rupture of an aromatic heterocycle nearby the Ni atoms. The formation of CN groups breaks the integrity of conjugated plane (Supplementary Figs. 2 and 3), which reduces electron migration and thus confines electron to the vicinity of the Ni atoms (Fig. 1c). The larger local electron density at the Ni site turns out to be beneficial for CO_2_RR, as explained as follows. Figure 1d and Supplementary Fig. 4 summarize the free energy diagrams for the transformation of CO_2_ into CO for all the systems mentioned above (i.e., the metal site, the support, the CN-modified support and combination of the metal site and modified support). It can be realized that overall, Ni@C_3_N_4_-CN shows the lower activation barriers for every intermediate step. Especially, it was predicted that Ni@C_3_N_4_-CN lowered the most the activation barrier of the initial step, hydrogenation of CO_2_, which turned out to be the rate-limiting step of the reaction (Fig. 1e and Supplementary Fig. 4): Ni@C_3_N_4_-CN (0.62 eV), Ni@C_3_N_4_ (0.70 eV), bare C_3_N_4_ (2.23 eV) and C_3_N_4_-CN (1.53 eV). When the -CN locates away from Ni sites (Ni@C_3_N_4_-CN-2), the adjacent π-conjugated aromatic heterocycles near Ni site keep intact resulting in no attenuation of *d*-π conjugation and thus the breaking of the conjugated plane no longer lowers the energy barrier of *COOH formation (Supplementary Fig. 5). On the contrary, −CN only works as electron withdrawing group to reduce the local electron density at the Ni site and thus the CO_2_RR performance.

In order to further evaluate the performance, the projected density of states (PDOS) (Supplementary Fig. 6) reveals that the *d*-band center (*ɛd*) is closer to the Fermi energy level (EF) for Ni@C_3_N_4_-CN, demonstrating the improved adsorption ability of CO_2_ and *COOH. Accompanying the larger shift of the *d*-band center after *COOH formation of Ni@C_3_N_4_-CN (0.673 eV) than that of Ni@C_3_N_4_ (0.230 eV), more electrons in the 3*d* orbital of Ni@C_3_N_4_-CN can be used to stabilize *COOH. To demonstrate the universality of this strategy, DFT calculations on other coordination numbers are conducted. Supplementary Fig. 5 exhibits that Ni@C_3_N_4_-CN 3-fold has a lower energy barrier of *COOH (0.14 eV) than that of Ni@C_3_N_4_ 3-fold (0.50 eV), demonstrating this strategy is also appropriate for other coordination number. These results suggest that by tailoring the metal-support interaction, the charge transfer processes can be modulated, resulting in an improved electrocatalytic activity of the binary system^15^.

### Catalyst synthesis and characterization

**Fig. 2:**
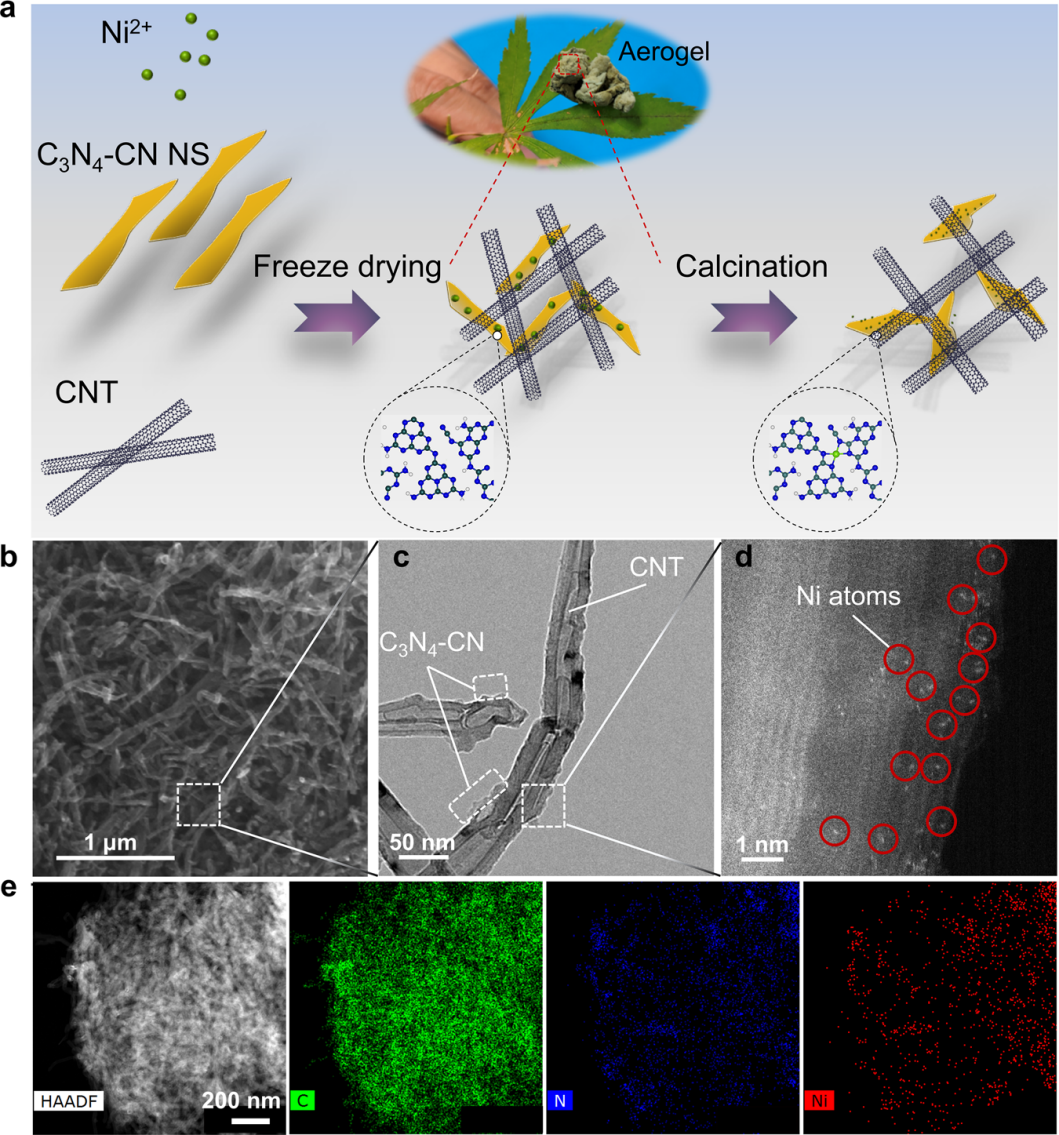
Physical characterization of Ni@C_3_N_4_-CN. **a** Schematic illustration of the preparation. **b** SEM image. **c** HRTEM image. **d** AC HAADF-STEM image. **e** EDS mapping image.

To verify the theoretical results, atomically dispersed Ni@C_3_N_4_-CN and Ni@C_3_N_4_ were prepared on carbon nanotubes (CNTs) (Scheme in Fig. 2a). C_3_N_4_ nanosheets decorated with either cyano groups (C_3_N_4_-CN NS) or hydroxylated (C_3_N_4_-OH NS) – used here as controls – were successfully prepared by salt-assisted or alkaline-assisted exfoliation methods^16,17^, respectively (Supplementary Figs. 7 and 8). The characterization shows that C_3_N_4_-CN and C_3_N_4_-OH nanosheets present a similar morphology, microstructure and dispersion ability in water (Supplementary Figs. 9–15). The nanosheets were further modified by depositing Ni atoms. The corresponding aerogels were obtained by a simple electrostatic self-assembly of C_3_N_4_-CN and C_3_N_4_-OH NS during freeze drying, which is done to promote the isolate adsorption of nickel ions and thus the synthesis of Ni single atom catalysts (SACs) (Supplementary Fig. 16). Finally, the Ni@C_3_N_4_-CN catalyst was synthesized through a facile calcination method.

Once synthesized, we continued with a thorough characterization of the SACs, which allowed us to gain understanding on the chemical and electronic properties of the catalysts. For instance, any Ni XRD signal can be detected for Ni@C_3_N_4_-CN and Ni@C_3_N_4_ but only the diffraction peaks of CNT substrate are visible, indicating the absence of metallic phases and suggesting a good dispersion of the Ni atoms (Supplementary Fig. 17). No metal contamination was found in these precursors (Supplementary Fig. 18). The Ni content in Ni@C_3_N_4_-CN and Ni@C_3_N_4_ was estimated to be 1.08 wt% and 1.17 wt%, respectively, as reveled by inductively coupled plasma optical emission spectrometer (ICP-OES).

Both the morphology and the atomic Ni dispersion was investigated through scanning electron microscope (SEM) (Supplementary Fig. 19), high-resolution transmission electron microscope (HRTEM) and aberration-corrected high-angle annular dark-field scanning transmission electron microscopy (AC HAADF-STEM) The results confirmed the single-atom nature of the Ni sites for both CNT-supported C_3_N_4_ and Ni@C_3_N_4_-CN, (Fig. 2b–e and Supplementary Figs. 20–23).

### Fine structure of Ni@C_3_N_4_-CN

**Fig. 3:**
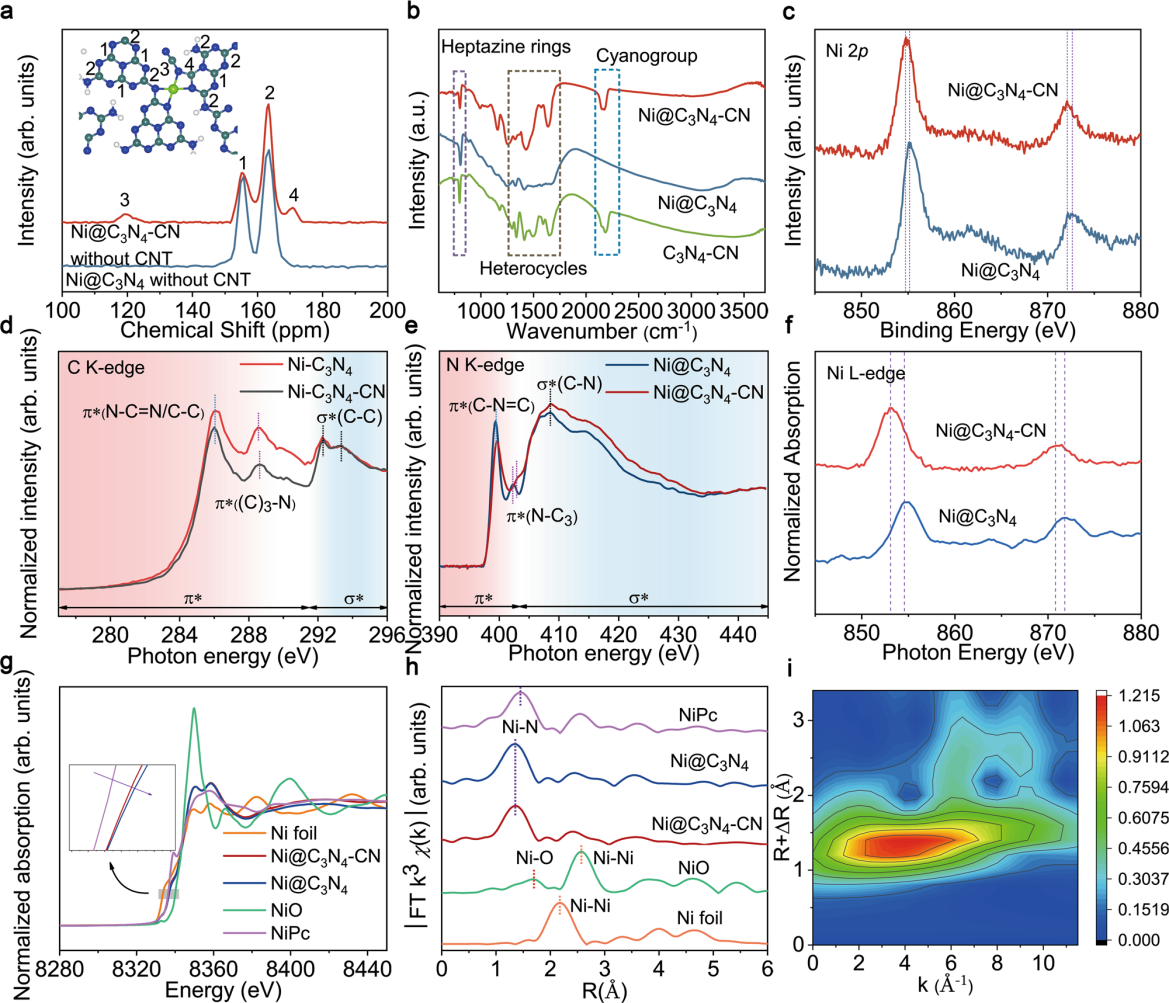
Electronic structure characterization of catalysts. **a** Solid-state ^13^C MAS NMR spectra of Ni@C_3_N_4_-CN, Ni@C_3_N_4_ without CNT. **b** FT-IR spectra of Ni@C_3_N_4_-CN, Ni@C_3_N_4_ and C_3_N_4_-CN catalyst. **c** High-resolution XPS of Ni 2*p* spectra. **d** XANES spectra of C K-edge. **e** XANES spectra of N K-edge. **f** XAS spectra of Ni L-edge. **g** Ni K-edge of Ni@C_3_N_4_-CN and Ni@C_3_N_4_. **h** k^3^ weighted Fourier transform spectra from EXAFS of Ni@C_3_N_4_-CN and Ni@C_3_N_4_. **i** WT-EXAFS plot for Ni@C_3_N_4_-CN.

To acquire the structural information of catalysts, solid-state ^13^C MAS NMR, Fourier transform infrared (FT-IR) and high-resolution X-ray photoelectron spectroscopy (XPS) spectra were performed. Solid-state ^13^C MAS NMR spectra of Ni@C_3_N_4_ show two strong peaks at 156.5 and 163.3 ppm, that correspond to the chemical shifts of C−N_3_ (1) and N_2_ − C−NH_x_ (2) in the aromatic heterocycles, respectively (Fig. 3a and Supplementary Fig. 11)^18^. Nevertheless, two new peaks at 171.0 and 120.4 ppm can be clearly observed for Ni@C_3_N_4_-CN, which can be ascribed to the C atom (4) and the −CN (3) directly attached to N coordination atoms of Ni sites, respectively^19^. As expected, the presence of −CN group in the support was also confirmed by FT-IR spectroscopy. The characteristic CN peak at 2180 cm^−1^, was observed for cyano functionalized substrates, unlike the substrate in which the functional group was absent (Fig. 3b)^20^.

We also studied the system by X-ray Photoelectron Spectroscopy (XPS), which not only enabled us to detect the presence of the cyano groups, but also the bond created between the N atoms of cyano groups and the Ni single atoms. N 1 *s* of C_3_N_4_-CN changed obviously after the addition of Ni sites, compared to C 1 *s*, indicating Ni sites coordinate with N atoms of C_3_N_4_ (Supplementary Fig. 24). Four peaks at about 401.2, 400.4, 399.6 and 398.5 eV, deconvoluted from N 1 *s* can be assigned to the N atoms in the surface amino groups, −CN, tri-coordinated N (N − (C)_3_) and two-coordinated N (C−N=C), respectively^21–23^. The ratio of tri-coordinated N (N_3_) to two-coordinated N (N_2_) (N_3_:N_2_) changed from 0.13 to 0.27 after Ni introduction, revealing Ni sites bind with the two-coordinated N in C_3_N_4_-CN^24^. Figure 3c shows the presence of Ni in both samples, as detected by XPS.

Next, we focused on the differences on the electronic properties between the substrates. Synchrotron-based X-ray adsorption near-edge structure (XANES) spectra allowed us to demonstrate that attenuating *d*-π conjugation is beneficial to electronic localization on Ni sites (Fig. 3d, e). The double peaks peak at 292.3 eV and 293.3 eV can be assigned to the C − C σ* states of CNT^25^. A strong peak appeared at 408.5 eV of Ni@C_3_N_4_-CN, corresponding to electron transition from the N 1 *s* to C−N σ* orbital due to the introduction of −CN^24^. Remarkably, the intensities of π*(N−C=N) (286.0 eV), π*((C)_3_ − N) (288.6 eV), π*(C−N=C) (399.5 eV) and π*(N − (C)_3_) (402.9 eV) for Ni@C_3_N_4_-CN were weaker than that of Ni@C_3_N_4_, revealing that the introduction of −CN weaken the π-conjugation and thus *d*-π conjugation^26^. Noticeably, the binding energy of Ni 2*p* and XAS spectra of the Ni L-edge (Fig. 3c, f) both have a negative shift in Ni@C_3_N_4_-CN compared with that of the Ni@C_3_N_4_, indicating the electronic localization of Ni sites in Ni@C_3_N_4_-CN^8^. Meanwhile, XANES spectra of Ni K-edge (Fig. 3c) shows a negative shift in the Ni@C_3_N_4_-CN compared to that of the Ni@C_3_N_4_ (Fig. 3g), demonstrating the electronic localization of Ni sites in Ni@C_3_N_4_-CN as well.

Fourier transformed (FT) extended X-ray absorption fine structure (EXAFS) manifests the atomic dispersion features of the Ni atoms in Ni@C_3_N_4_-CN and Ni@C_3_N_4_^27^. Both of them exhibit a coordination number of ~4, which was obtained from well-fitting process (Supplementary Fig. 25 and Table 1). Wavelet transform (WT) EXAFS with high resolution in both k and R space demonstrates the existence of Ni−N_4_ configuration in catalysts (Fig. 3i and Supplementary Fig. 26)^28^. Thus, these results reveal the experimental structures are identical as the computational ones and attenuating *d*-π conjugation promotes electronic localization on Ni sites.

### Evaluating catalyst performance for CO_2_RR

**Fig. 4:**
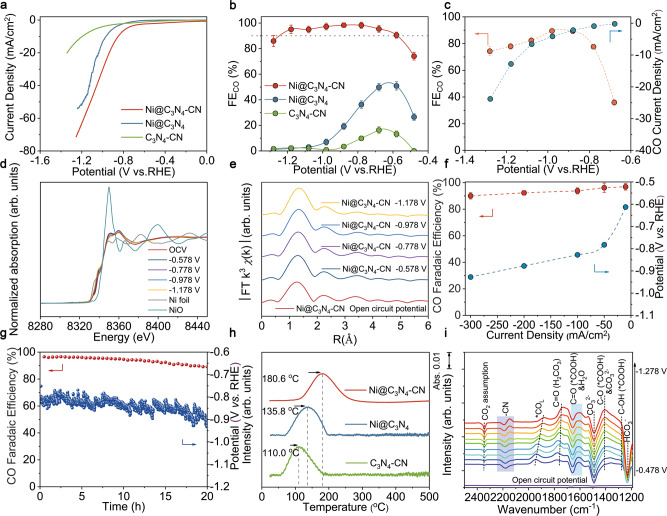
Electrochemical CO_2_RR performances. **a** LSV curves at scan rate of 10 mV/s in H-cell with pure CO_2_ saturated 0.5 M KHCO_3_ solution. **b** FE_CO_ at different potentials in H-cell under pure CO_2_. **c** FE_CO_ and *J*_*CO*_ of Ni@C_3_N_4_-CN at different potentials under 30% CO_2_ concentration. **d** In situ XANES spectra of Ni@C_3_N_4_-CN measured at different potentials. **e** In situ k^3^ weighted Fourier transform EXAFS spectra of Ni@C_3_N_4_-CN. **f** The potentials and FE_CO_ at different current densities of Ni@C_3_N_4_-CN in flow cell under pure CO_2_. **g** Stability of Ni@C_3_N_4_-CN at a current density of −100 mA/cm^2^ in flow cell under pure CO_2_. **h** CO_2_ TPD curves of Ni@C_3_N_4_-CN, Ni@C_3_N_4_, C_3_N_4_-CN catalyst. **i** In situ ATR-IR spectra of Ni@C_3_N_4_-CN. The error bars correspond to the standard deviations of measurements over three separately prepared samples under the same testing conditions.

To evaluate the performance of Ni@C_3_N_4_-CN, electrochemical tests were conducted (Supplementary Fig. 27). According to gas chromatograph (GC) and ^1^H-NMR spectra, no C_2_ and liquid products was observed (Supplementary Figs. 28 and 29). ^13^CO_2_ was used as the feedstock to carry out the electrolysis test in the KHCO_3_ electrolyte, confirming CO is the conversion product of CO_2_ (Supplementary Fig. 30). Ni@C_3_N_4_-CN has larger current densities than those of Ni@C_3_N_4_ and C_3_N_4_-CN catalysts (Fig. 4a). Moreover, Ni@C_3_N_4_-CN is selective to CO production with Faradaic efficiency of CO (FE_CO_) ≥ 90% over a wide potential range from −0.578 to −1.178 V vs. RHE and the maximal FE_CO_ could reach ~99% (Fig. 4b). Remarkably, the maximal *J*_*CO*_ and TOF value with a FE_CO_ ≥ 90% of Ni@C_3_N_4_-CN could attain 46.8 mA/cm^2^ and ~22,000 h^−1^ at −1.178 V vs. RHE, which are far superior to those of Ni@C_3_N_4_ (0.82 mA/cm^2^, ~410 h^−1^) (Fig. 4b and Supplementary Fig. 31 and 32). Ni@C_3_N_4_-CN is a promising electrocatalyst for CO_2_ reduction to CO compared with other recently published works when considering *J*_*CO*_, TOF and the potential window in KHCO_3_ electrolyte under conditions of FE_CO_ ≥ 90% (Supplementary Fig. 35). The in situ XAS was conducted to demonstrate that the single Ni atoms don’t aggregate under CO_2_ reduction (Fig. 4d, e and Supplementary Fig. 33). To assess the stability of the catalyst, FE_CO_ and *J*_*CO*_ were monitored during 12 h of electroreduction at −0.878 V vs. RHE in H-cell (Supplementary Fig. 34). To investigate the performance in practical application, the performance low CO_2_ concentrations in H-cell were acquired^29^. Ni@C_3_N_4_-CN retains a FE_CO_ above 90% at −0.878 and −0.978 V vs. RHE, even when the CO_2_ concentration was reduced to 30% (Fig. 4c).

To further assess the potential of the Ni@C_3_N_4_-CN catalyst for industrial applications, the flow cell was assembled. Ni@C_3_N_4_-CN shows a current density of −300 mA/cm^2^ with a CO Faradaic efficiency ≥90% and an energy efficiency (EE) of 70.4% at −0.61 V under pure CO_2_ (Fig. 4f and Supplementary Fig. 36, Table 2). Furthermore, the flow cell also shows −100 mA/cm^2^ while maintaining a FE_CO_ ≥ 90% and the single pass conversion (SPC) could reach 11.23% under 30% CO_2_ concentration (Supplementary Fig. 37 and Table 3). The CO_2 crossover_ only reached 20.3% and 16.8% under pure and 30% CO_2_ concentrations, respectively. The flow cell could achieve ~20 h stability with a FE_CO_ above 90% under both pure and 30% CO_2_ concentration (Fig. 4g and Supplementary Figs. 37 and 38). Thus, Ni@C_3_N_4_-CN catalyst exhibits a desirable prospect in practical application.

To analyze the CO_2_ activation process, EIS (Electrochemical impedance spectroscopy) studies were carried out (Supplementary Fig. 39)^30^. Ni@C_3_N_4_-CN have a lower charge-transfer resistance value in the electro-proton transfer steps than that of Ni@C_3_N_4_, suggesting a fast charge–transfer capacity (CO_2_ → *COOH and *COOH → *CO) for Ni@C_3_N_4_-CN^9^. Since the conversion of *COOH to *CO is generally considered a thermodynamically downhill process, the result of EIS shows Ni@C_3_N_4_-CN has a better CO_2_ activation ability than that of Ni@C_3_N_4_-CN. To deeply explore the ability of CO_2_ activation on Ni sites, CO_2_ TPD and electrochemical activation test were conducted. Ni@C_3_N_4_-CN shows a stronger CO_2_ adsorption signal of CO_2_ adsorption, compared with that of bare C_3_N_4_-CN and Ni@C_3_N_4_, demonstrating the facile CO_2_ activation on Ni sites of Ni@C_3_N_4_-CN (Fig. 4h). Furthermore, OH^−^ was chosen as a substitution to simulate the CO_2_ activation process through the oxidative LSV scans in N_2_-saturated 0.5 M NaOH electrolyte (Supplementary Fig. 40)^31^. As a result, the potential for surface OH^−^ adsorption on Ni@C_3_N_4_-CN is more negative than that on Ni@C_3_N_4_, implying the stronger ability of CO_2_ adsorption and thus activation through attenuating *d*-π conjugation. Therefore, ex-situ characterizations show Ni@C_3_N_4_-CN has a better potential in CO_2_RR than Ni@C_3_N_4_ in the matter of CO_2_ activation.

In situ ATR-IR measurements were employed to study real-time intermediates on metal sites during CO_2_RR to CO (Supplementary Fig. 41)^32^. Peaks located at around 2343 cm^−^^1^and range from 1894 cm^−^^1^ to 1950 cm^−1^ can be attributed to CO_2_ assumption and *CO (adsorbed linear-bonded CO) respectively (Fig. 4i and Supplementary Fig. 42)^33^. There are many overlaps among the peaks of H−O−H bending, HCO_3_^−^/CO_3_^2−^ and *COOH intermediate, which makes it difficult to directly analyze the peak of *COOH intermedia (Supplementary Table 4). Hence, in order to avoid the effect of H−O−H bending around 1580–1650 cm^−^^1^, we used D_2_O to prepare electrolyte for in situ ATR-IR. Noticeably, as shown in Supplementary Fig. 43, the obvious peak of C=O (*COOH) stretching appears in ~1580–1620 cm^−^^1^. The intensity of C=O (*COOH) and C−O (*COOH) stretching in Ni@C_3_N_4_-CN is much larger than that of Ni@C_3_N_4_, indicating the facilitated *COOH formation after attenuating *d*-π conjugation. Notably, the bands of −CN (the region a) appeared blue shifted (triple bond shortened) during CO_2_ activation (Supplementary Fig. 44), consistent with the result of DFT calculation. Because the change of −CN shares a same magnitude with those of the intermediates *COOH or *CO, the blue shifts of −CN are attributed to the inductive effect after *COOH or *CO adsorption on the adjacent Ni sites, proving the fine structure of Ni@C_3_N_4_-CN as the previous results of EXAFS. Thus, the results of TPD, electrochemical analytical test and in situ ATR-IR confirm that Ni@C_3_N_4_-CN exhibits a more favorable CO_2_ activation pathway than that of Ni@C_3_N_4_, which is consistent with the results of CO_2_RR performance and DFT calculations.

In summary, we demonstrated the influence of electron density manipulation at the single atom scale for CO_2_RR. DFT calculations prove that attenuating *d*-π conjugation is beneficial for CO_2_ activation on Ni sites due to the reduced charge migration from metal atoms towards the substrate in the presence of the –CN functional groups. Then, we successfully synthesized Ni SACs with attenuated *d*-π conjugation through the introduction of –CN moieties, Ni@C_3_N_4_-CN. Comprehensive characterizations reveal the specific fine structure of Ni single atom sites in Ni@C_3_N_4_-CN and its respective control, Ni@C_3_N_4_. Ni@C_3_N_4_-CN as an electrocatalyst for CO_2_ reduction to CO exhibits a TOF of ~22,000 h^−1^ in H-cell, and maintains a FE_CO_ of over 90% even under a realistic CO_2_ concentration of only 30%. Moreover, Ni@C_3_N_4_-CN incorporated in flow cell exhibits a large current density (−300 mA/cm^2^) with a FE_CO_ ≥ 90%. Finally, CO_2_ TPD, electrochemical activation test and in situ ATR-IR demonstrate the easier CO_2_ activation on Ni@C_3_N_4_-CN, which is in good accordance with the DFT results. Our work offers a new insight in the design of atomically dispersed metal sites for CO_2_ activation in CO_2_RR. Engineering electrocatalysts with atomic precision and fine-tuning of its electronic properties is crucial towards controlling multi-electron reduction processes. As demonstrated in here, the modulation of the charge transfer within components of the catalyst is one of the key variables to be considered when designing and synthesizing future SACs towards CO_2_RR.

## Methods

### Chemicals

Dicyandiamide (DCDA), NaCl, KCl, and NiCl_2_ were bought from Shanghai Aladdin reagent co. Ltd. Carboxylated multiwalled carbon nanotube (CMWCNT, 30–50 nm in diameter) was purchased from Pioneer Nanotechnology Co. Ltd. All the chemical reagents except CMWCNT were used as received without any other purification. Before using the CMWCNT, 0.5 M HNO_3_ was used to remove the potential metal impurities at 80 °C for 12 h.

### Synthesis of C_3_N_4_ Bulk

A classic method was used for the synthesis of C_3_N_4_ bulk. 0.07 mol DCDA was added in a 50 mL covered crucible and then heated in a muffle furnace at 550 °C with a 5 °C/min heating rate and then retained at 550 °C for 120 min.

### Synthesis of C_3_N_4_-OH nanosheets (C_3_N_4_-OH NS)

Alkaline-assisted exfoliation method was used for the synthesis of C_3_N_4_-OH nanosheets. Briefly, the obtained yellow C_3_N_4_ bulk was ground into powder by an agate mortar. Then C_3_N_4_ bulk powder (500 mg) was mixed with 20 mL NaOH solution (1 M) in a plastic beaker. The mixture was stirred at 60 °C for 12 h. Then the mixture was transferred to a dialysis bag (MD55-3500) to remove excess NaOH by dialysis (dialysis bag, MD55-3500) until neutral (about 6 days). Finally, the white C_3_N_4_-OH nanosheets powder were obtained by rotary evaporation at 60 °C.

### Synthesis of C_3_N_4_-CN nanosheets (C_3_N_4_-CN NS)

Salt-assisted method was used for the synthesis of C_3_N_4_-CN nanosheets. 0.015 mol NaCl, 0.015 mol KCl and 0.07 mol DCDA were grinded evenly and packed in a 50 mL covered crucible. The crucible was wrapped by tinfoil and then heated in a muffle furnace at 670 °C with a 2 °C/min heating rate and then retained 670 °C for 45 min. The mixture after pyrolysis was dissolved in a solution in which the volume ratio of deionized (DI) water to ethanol is 2:1. Then the centrifugation was conducted to remove NaCl and KCl solution preliminarily. For further purification, the mixture was transferred to a dialysis bag (MD55-3500). Dialysis lasted for 7 days. Finally, the brown C_3_N_4_-CN nanosheets powder were obtained by rotary evaporation at 60 °C.

### Synthesis of Ni@C_3_N_4_ catalyst

A mixture of 0.02 g C_3_N_4_-OH nanosheets and 0.008 g CMWCNT were added to 30 mL DI water. C_3_N_4_-OH nanosheets were spread out and shattered through 60 min ultrasound. 0.05 mL of 0.1 M NiCl_2_ was added dropwise to the mixture while stirring for 2 h. The liquid nitrogen was poured directly into the mixed solution to obtain an ice block. After the ice block was freeze-dried for 72 h, the gray aerogel was obtained. The aerogel was heated to 600 °C with a 5 °C/min heating rate at Ar atmosphere, without heat preservation, followed by cooling to room temperature immediately.

### Synthesis of Ni@C_3_N_4_-CN catalyst

A mixture of 0.02 g C_3_N_4_-CN nanosheets and 0.008 g CMWCNT were added to 30 mL DI water. C_3_N_4_-CN nanosheets were spread out and shattered through 60 min ultrasound. 0.05 mL of 0.1 M NiCl_2_ was added dropwise to the mixture while stirring for 2 h. The liquid nitrogen was poured directly into the mixed solution. Then the aerogel was obtained after the dark green ice block was freeze-dried for 72 h. The aerogel was heated to 600 °C with a 5 °C/min heating rate at Ar atmosphere, without heat preservation, followed by cooling to room temperature immediately.

### Synthesis of C_3_N_4_-CN catalyst

The synthesis of C_3_N_4_-CN catalyst was same as Ni@C_3_N_4_-CN except that NiCl_2_ was not added.

### Characterizations

Solid state ^13^C nuclear magnetic resonance (NMR) was measured on an Agilent 600 M spectrometer. The Fourier transform infrared (FT-IR) spectra were obtained on a Nicolet iS50 FT-IR spectrometer. Powder X-ray diffraction (XRD) patterns were collected by using a D8 advance X-ray diffractometer (Rigaku, Japan) with Cu Kα radiation (λ = 0.15406 nm) at a scan rate (2θ) of 10 °C/min. The morphologies of the samples were determined by Field emission scanning electron microscopy (SEM, Hitachi S-4800) and high-resolution transmission electron microscopy with a spherical aberration corrector (HRTEM, Titan G2 60-300) equipped with energy dispersive X-ray spectroscopy (EDS) mapping. The atomically dispersed metal atoms were detected by Aberration-corrected HAADF-STEM (JEM-ARM200F). C, N, Ni X-ray absorption spectra were obtained at beamlines 01C1 of the National Synchrotron Radiation Research Center (NSRRC, Taiwan). X-ray photoelectron spectroscopy (XPS) measurements were performed on Thermo Fisher Scientific Escalab 250 XI, and all the binding energies were calibrated by the C 1 *s* peak at 284.8 eV. The BET specific surface areas were obtained from JW-BK200C nitrogen sorption analyzer (Beijing JWGB SCI. & Tech. Co., Ltd) with 150 °C pretreatment in high vacuum, and the pore size distribution was calculated from the adsorption branch of the isotherms. Raman spectra were obtained by a DXRI Raman Microscope (Thermo Fisher) using a 532 nm laser as the light source. CO_2_ and CO temperature program desorption (TPD) curves were measured on Micromeritics AutoChem 2920. Inductively Coupled Plasma Mass Spectrometry (ICP-MS, Agilent 7700 s) was used to measure the content of metal atoms in the samples. The gas phase products after bulk electrolysis were quantified by on-line Gas chromatograph (GC, Shimidzu, Model 2014).

### Electrochemical measurements

All electrochemical measurements in this study were implemented with an electrochemical station of Auto Lab in a typical three electrode system. The customized gas-tight H-cell, with a conventional three electrode system, comprised carbon paper with catalysts coating, Ag/AgCl reference electrode (3.5 M KCl) and Pt mesh counter electrode. An anion exchange membrane (Nafion-117) was used to separate these two compartments. 1 mg catalyst was mixed with 970 μL isopropanol and 30 μL Nafion solutions (5 wt%, Sigma-Aldrich) followed by sonication of 30 min to form a homogeneous solution. The as-obtained catalyst ink was dropped onto a carbon paper (0.25 cm^2^) directly and dried at 70 °C for 8 h. The mass loading of the catalyst was 0.2 mg/cm^2^. All potentials were referenced to reversible hydrogen electrode (RHE) with the formula of E (RHE) = E (Ag/AgCl) + 0.205 V + 0.059 V × pH after iR compensation. The electrolyte was 0.5 M KHCO_3_ (30 mL for each compartment) and saturated with high purity CO_2_ (99.999%) for at least 30 min before testing (20 sccm, calibrated by mass flow controller). LSV curves were collected with the scan rate of 10 mV/s. Constant potential electrolysis was carried out at various potentials for 20 min to analyze the products. The uncompensated solution resistance was compensated for 95% by EIS measurement which were conducted from 100 kHz to 0.1 Hz.

The cathodic products were analyzed by an on-line gas chromatograph. High-purity N_2_ (99.999%) was used as the carrier gas. A TCD was used to measure the H_2_ fraction and a flame ionization detector was equipped with a nickel conversion furnace to analyze the CO fraction. The Faradaic efficiency of products was calculated from gas chromatograph chromatogram peak according to the following equation:

1 $${{{{{{\rm{FE}}}}}}}_{{{{{{\rm{CO}}}}}}\;{{{{{\rm{or}}}}}}\;{{{{{{\rm{H}}}}}}}_{2}}=x\times V\times \frac{2{{{{{\rm{F}}}}}}{{{{{{\rm{P}}}}}}}_{0}}{i{{{{{\rm{RT}}}}}}}$$

$$x$$: fraction value

*V*: flow rate of CO_2_

${{{{{\rm{F}}}}}}$: faraday constant (96485 C/mol)

${{{{{{\rm{P}}}}}}}_{0}$: normal atmosphere (101325 Pa),

*I*: applied current,

${{{{{\rm{R}}}}}}$: gas constant (8.314 J/(mol·K))

$${{{{{\rm{T}}}}}}$$: room temperature (298 K).

#### TOF calculations

We calculate the TOF according to the following equation:

2 $${{{{{\rm{TOF}}}}}}({{{{{{\rm{h}}}}}}}^{-1})=\frac{{I}_{{product}}/{{{{{\rm{nF}}}}}}}{{m}_{{cat}}\times \alpha /{{{{{{\rm{M}}}}}}}_{{{{{{\rm{metal}}}}}}}}\times {3600}$$

*I*_*product*_*:* partial current for CO, A

n: number of electrons transferred for CO, 2

F: Faradaic constant, 96485 C/mol

*m*_*cat*_: catalyst mass in the electrode, g

*α*: mass ratio of active atoms in catalysts

M_metal_: atomic mass of metal

#### Cathodic EE calculations

3 $${{{{{\rm{EE}}}}}}(\%)=100\%\times \frac{1.23-{{{{{{\rm{E}}}}}}}_{0}}{1.23-{{{{{\rm{E}}}}}}}\times {{{{{\rm{FE}}}}}}\,(\%)$$

where E_0_, FE and E represented standard potential (CO, −0.11 V), faradaic efficiency and applied potential, respectively.

#### SPC of CO_2_ calculations at 25 °C, 1 atm

4 $${{{{{{\rm{CO}}}}}}}_{2 \, {{{{{\rm{consumed}}}}}}}({{{{{\rm{L}}}}}}\,{{{\min }}}^{-1})=	 \, (j\,{{{{{\rm{mA}}}}}}\,{{{{{{\rm{cm}}}}}}}^{-2})\left(\frac{1\,{{{{{\rm{A}}}}}}}{1000\,{{{{{\rm{mA}}}}}}}\right)\times \left(\frac{60\,{{{{{\rm{s}}}}}}}{1\,{{\min }}}\right)\times \left(\frac{1\,{{{{{\rm{mol}}}}}}\,{{{{{{\rm{e}}}}}}}^{-}}{96485\,{{{{{\rm{C}}}}}}}\right) \\ 	 \times \left(\frac{1 \, {{{{{\rm{mol}}}}\;{{{\rm{CO}}}}}}}{2 \, {{{{{\rm{mol}}}}}} \, {{{{{{\rm{e}}}}}}}^{-}}\right)\times \left(\frac{1 \, {{{{{\rm{mol}}}}}}\,{{{{{{\rm{CO}}}}}}}_{2}}{1 \, {{{{{\rm{mol}}}}\,{{{\rm{CO}}}}}}}\right)\times \left(\frac{24.05\ {{{{{\rm{L}}}}}}}{ 1\, {{{{{\rm{mol}}}}}}\,{{{{{{\rm{CO}}}}}}}_{2}}\right)\times (1 \, {{{{{{\rm{cm}}}}}}}^{2})$$

5 $${{{{{\rm{SPC}}}}}}\,(\%)=100\%\,\times \, \left(\frac{{{{{{{\rm{CO}}}}}}}_{2 \, {{{{{\rm{consumed}}}}}}}\,({{{{{{\rm{L}}}}}}}\,{{{\min }}}^{-1})}{{{{{{{\rm{CO}}}}}}}_{2 \, {{{{{\rm{flow}}}}\,{{{\rm{rate}}}}}}}\,({{{{{\rm{L}}}}}}\,{{{\min }}}^{-1})}\right)$$

where *j* is the partial current density CO production from CO_2_ reduction.

#### Calculation of CO_2_ cross-over

6 $${{{{{{\rm{CO}}}}}}}_{2\,{{{{{\rm{crossover}}}}}}}\,(\%)=100\%\times \left(\frac{{{{{{{\rm{CO}}}}}}}_{2\,{{{{{\rm{inlet}}}}}}}-{{{{{{\rm{CO}}}}}}}_{2\,{{{{{\rm{outlet}}}}}}}-{{{{{{\rm{CO}}}}}}}_{2\,{{{{{\rm{consumed}}}}}}}}{{{{{{{\rm{CO}}}}}}}_{2\,{{{{{\rm{inlet}}}}}}}}\right)$$

### In situ attenuated total reflection-infrared spectroscopy (ATR-IR)

ATR-IR was carried out on a Nicolet iS50 FT-IR spectrometer equipped with an MCT detector cooled with liquid nitrogen. The Au-coated Si semi-cylindrical prism (20 mm in diameter) was employed as the conductive substrate for catalysts and the IR refection element. The catalysts suspensions were dropped on the Au/Si surface as the working electrode. The mass loading of the catalyst was 1 mg/cm^2^ and the electrolyte was 0.5 M KHCO_3_. In situ ATR-IR spectra were recorded during stepping the working electrode potential.

### Assembly of flow cell

The flow cell measurements were performed on a home-made cell including sandwich of flow frames, gaskets and an anion-exchange membrane (Selemion DSVN). In the flow cell, 3 mg catalyst was mixed with 950 μL isopropanol, 150 μL PTFE solutions (Polytetrafluoroethylene, 1 wt%) and 50 μL Nafion solutions (5 wt%, Sigma-Aldrich) followed by sonication of 30 min to form a homogenous solution. The obtained catalyst ink was dropped onto gas diffusion electrodes (GDEs, SGL29BC) (3 cm^2^) directly and then dried at 70 °C for 8 h. The loading of catalyst is 1 mg/cm^2^ and the area contacting with electrolyte is 1 cm^2^. the IrO_2_–coating titanium sheet is used as counter electrode and an Ag/AgCl (with saturated 3.5 M KCl) electrode as a reference electrode. The flow rate of the electrolyte (1 M KHCO_3_) was set at 30 mL/min in both of cathodic and anodic chambers. The potentials at different current densities in flow cell were obtained after iR compensation.

### Computational methods

Density functional theory (DFT) calculations were employed by Vienna Ab initio Simulation Package (VASP) with the projector augment wave (PAW) method^34–38^. The exchange and correlation potentials were present in the generalized gradient approximation with the Perdewe-Burkee-Ernzerh of (GGA-PBE)^39^. To explore the reaction pathways of CO_2_ to CO, a supercell consisting of 72 atoms (Ni atoms in C_3_N_4_ was built). A vacuum slab with 15 Å was added onto the C_3_N_4_ and Ni atoms in C_3_N_4_ surface to avoid the interaction in-fluence of the periodic boundary conditions. Spin polarization was taken into account in all calculations. van der Waals (VDW) interactions were corrected using the D2 method of Grimme^40^. A Monkhorst-Pack mesh with 2 × 2 × 1 K-points was used for Brillouin zone integration. The energy cutoff, convergence criteria for energy and force were set as 450 eV, 10^−5^ eV/atom and 0.02 eV/Å, respectively.

The computational hydrogen electrode (CHE) model was used to calculate the free energy diagram^41–43^. The aqueous environment of the electrolyte was treated with a continuum dielectric model as implemented by the Hennig group in the VASP_solv_ code^44,45^. The reaction free energy (ΔG) was calculated as follows:

7 $$\varDelta G=\varDelta E+\varDelta {ZPE}-{{{{{\rm{T}}}}}}\times \varDelta S$$

where ΔE is the chemisorption energy calculated by the DFT method. *ΔZPE* and *ΔS* are the differences in zero-point energies and entropy during the reaction, respectively.

## Supplementary information

Supplementary Information

## Data Availability

Full data supporting the findings of this study are available within the article and its Supplementary Information, as well as from the corresponding author upon reasonable request.
