# Supplementary material for: Attenuating metal-substrate conjugation in atomically dispersed nickel catalysts for electroreduction of CO2 to CO
Source: Nat Commun. 2022 Oct 14;13:6082. doi: 10.1038/s41467-022-33692-0 (PMC9568552; doi:10.1038/s41467-022-33692-0)
Supplement: Supplementary file 1 — Supplementary Information [file 41467_2022_33692_MOESM1_ESM.pdf]

Supporting Information

Attenuating metal-substrate conjugation in atomically dispersed nickel catalysts for electroreduction of CO_2 to CO

Qiyou Wang[1], Kang Liu[1], Kangman Hu[1], Chao Cai[1], Huangjingwei Li[1], Hongmei Li[1], Matias Herran[2], Ying-Rui Lu[3], Ting-Shan Chan[3], Chao Ma[4], Junwei Fu[1], Shiguo Zhang[4], Ying Liang[5], Emiliano Cortés[2*] & Min Liu[1*]

[1]Hunan Joint International Research Center for Carbon Dioxide Resource Utilization, State Key Laboratory of Powder Metallurgy, School of Physics and Electronics, Central South University, Changsha 410083, China.

[2]Nanoinstitut München, Fakultät für Physik, Ludwig-Maximilians-Universität München, 80539 München, Germany.

[3]National Synchrotron Radiation Research Center, 300 Hsinchu, Taiwan.

[4]College of Materials Science and Engineering, Hunan University, 410082, China.

[5]College of Food Science and Engineering, Central South University of Forestry and Technology, 410004, China

[*]emails: Emiliano.Cortes@lmu.de, minliu@csu.edu.cn

Supplementary Figures

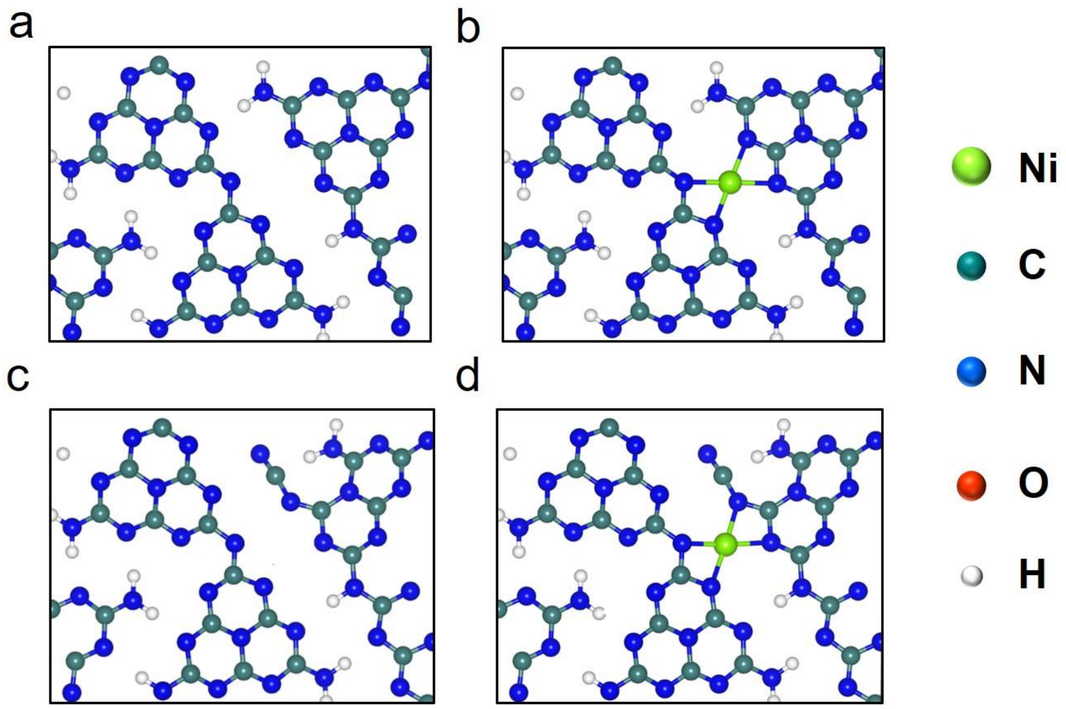

Supplementary Figure 1. The model of C_3N_4-OH, C_3N_4-CN, Ni@C_3N_4-CN and Ni@C_3N_4.

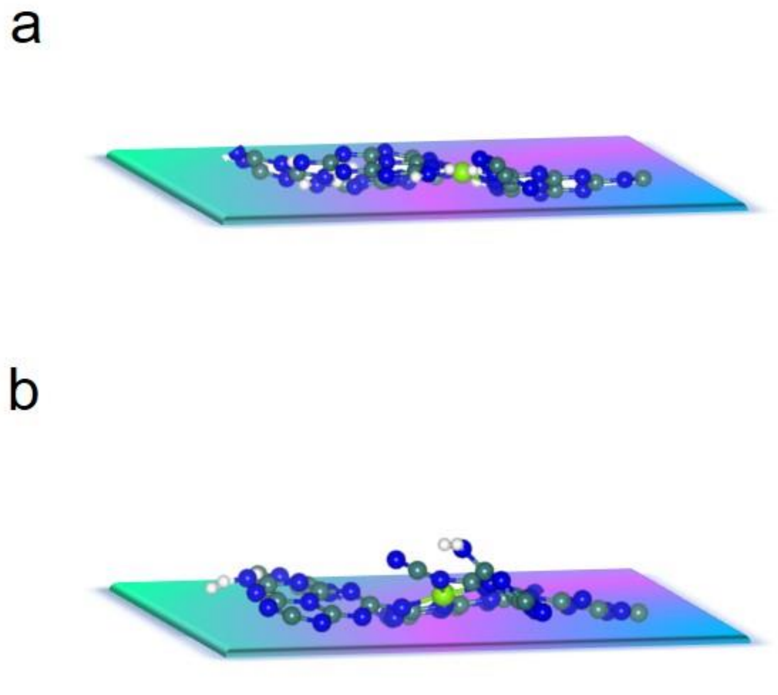

Supplementary Figure 2. Side view of Ni@C_3N_4-CN and Ni@C_3N_4.

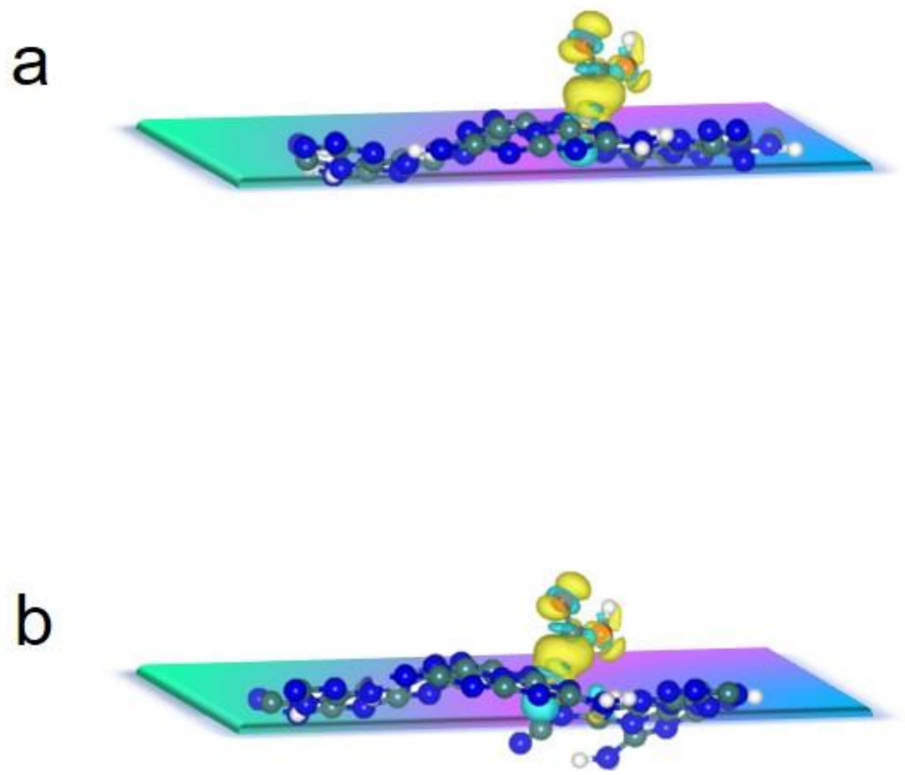

Supplementary Figure 3. The charge density differences COOH adsorbed on Ni@C_3N_4-CN and Ni@C_3N_4.

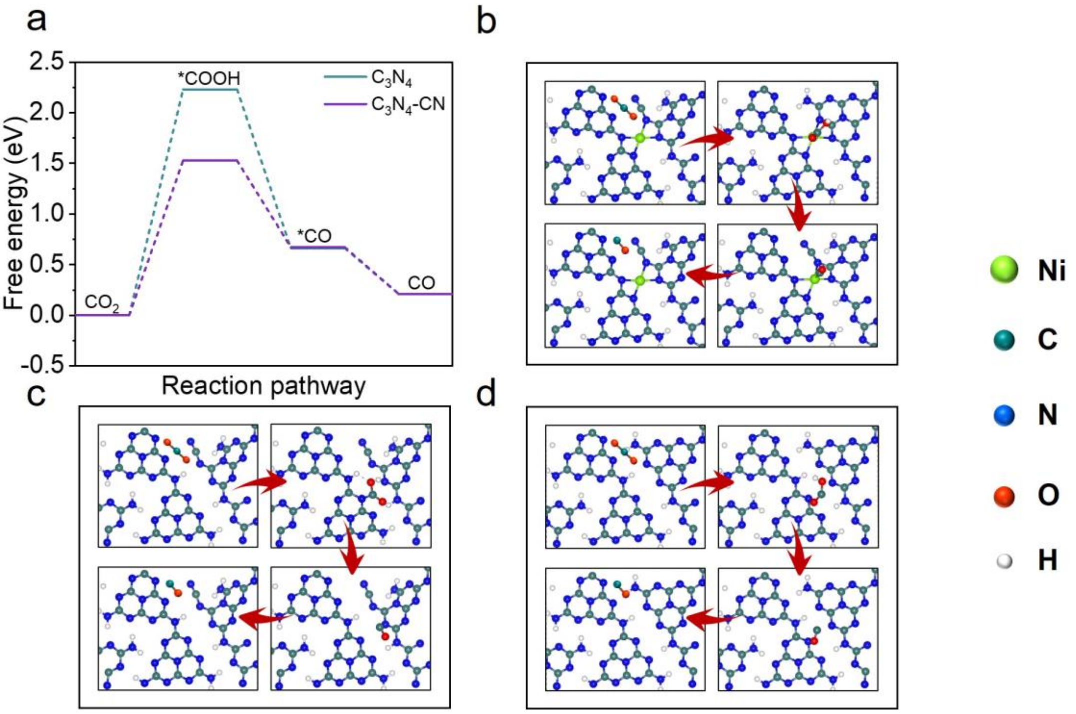

Supplementary Figure 4. a Free energy diagram of C_3N_4-CN and C_3N_4. Structure and adsorption configurations of key intermediates on **b** Ni@C_3N_4. **c** C_3N_4-CN. **d** C_3N_4.

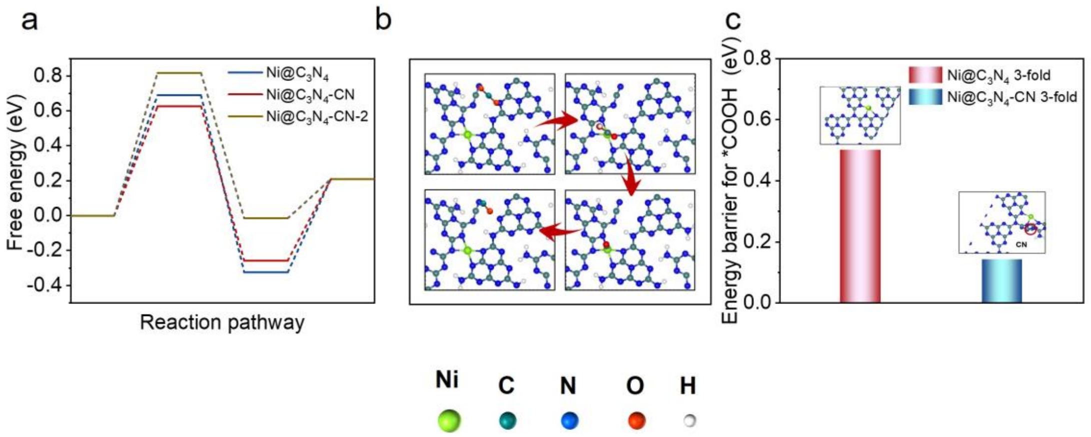

Supplementary Figure 5. a Free energy diagram of Ni@C_3N_4-CN, Ni@C_3N_4 and Ni@C_3N_4-CN-2. **b** Structure and adsorption configurations of key intermediates on Ni@C_3N_4-CN-2. **c** Energy barrier of *COOH formation for 3-fold coordination.

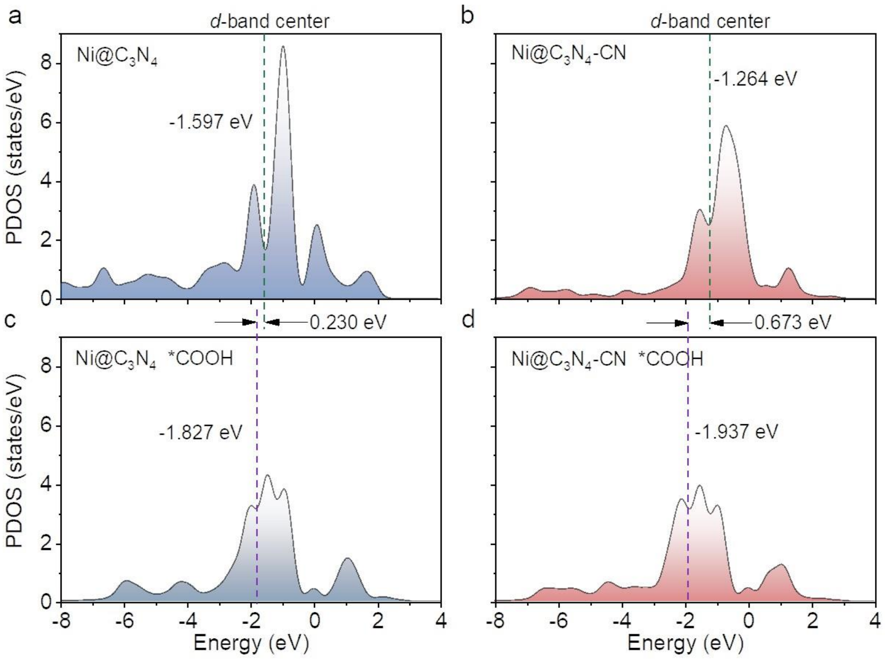

Supplementary Figure 6. Projected electron density of states (PDOS). a. Ni@C_3N_4. **b** Ni@C_3N_4-CN. **c** Ni@C_3N_4 interaction with COOH*. **d** Ni@C_3N_4-CN interaction with COOH*.

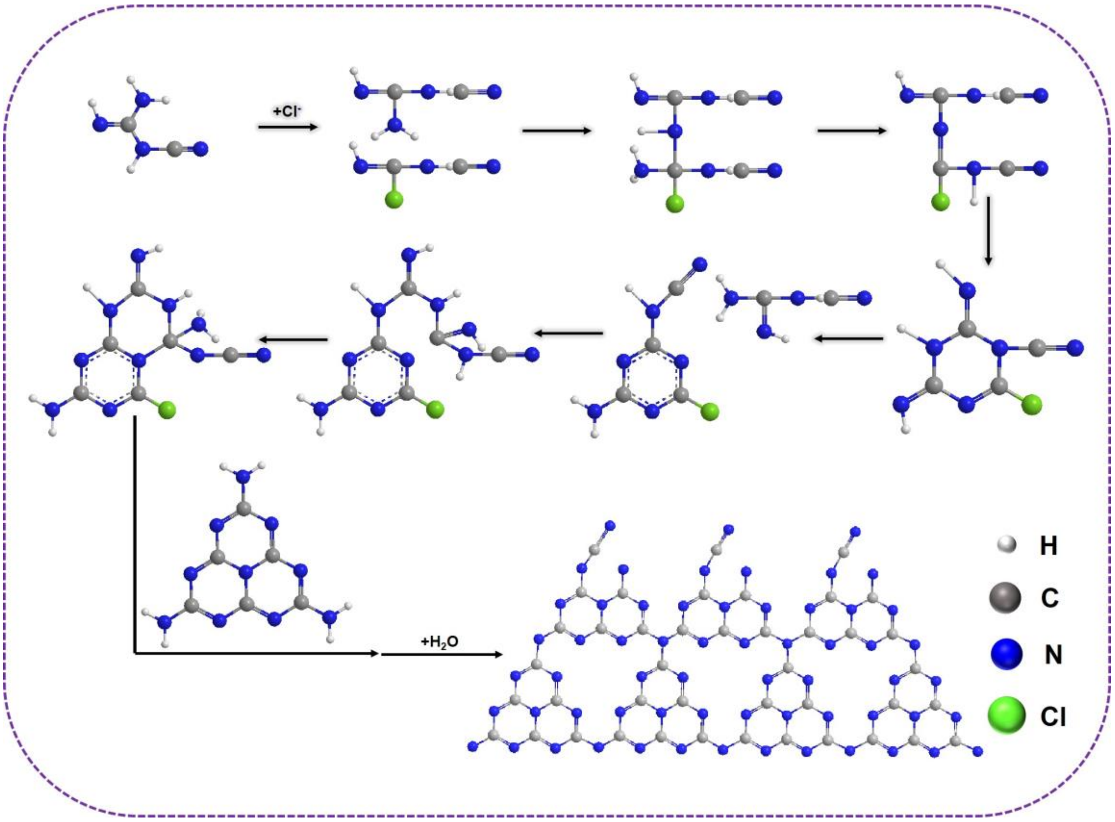

Supplementary Figure 7. The proposed formation mechanism of as-prepared C_3N_4-CN NS.

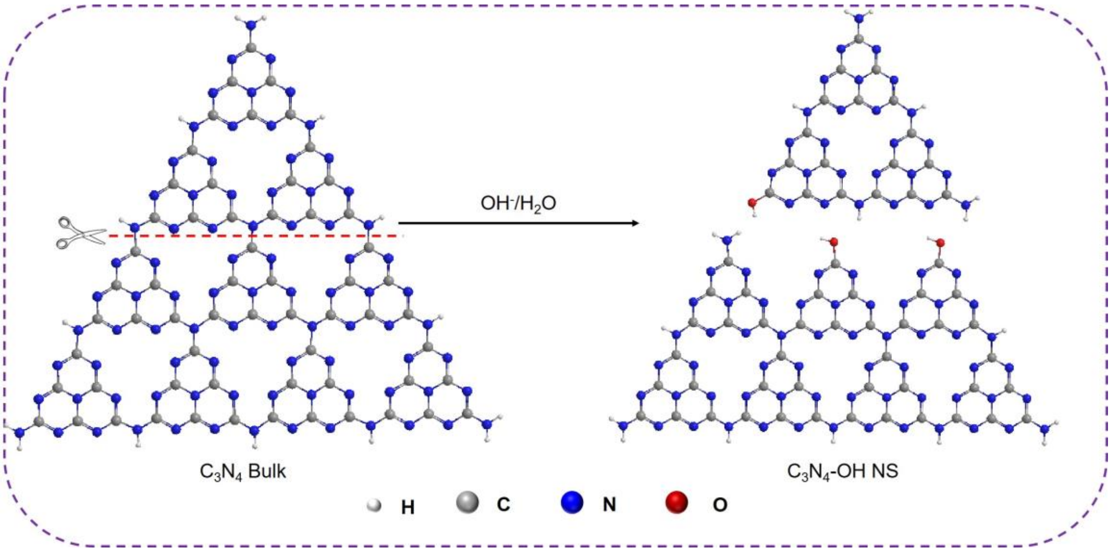

Supplementary Figure 8. The proposed formation mechanism of as-prepared C_3N_4-OH NS form C_3N_4 bulk. It was demonstrated in other works that the OH^- would work like a scissor tailoring the framework of C_3N_4 into smaller units and simultaneously modify edges with functional groups such as $-NH_x$ and $-OH$[1, 6].

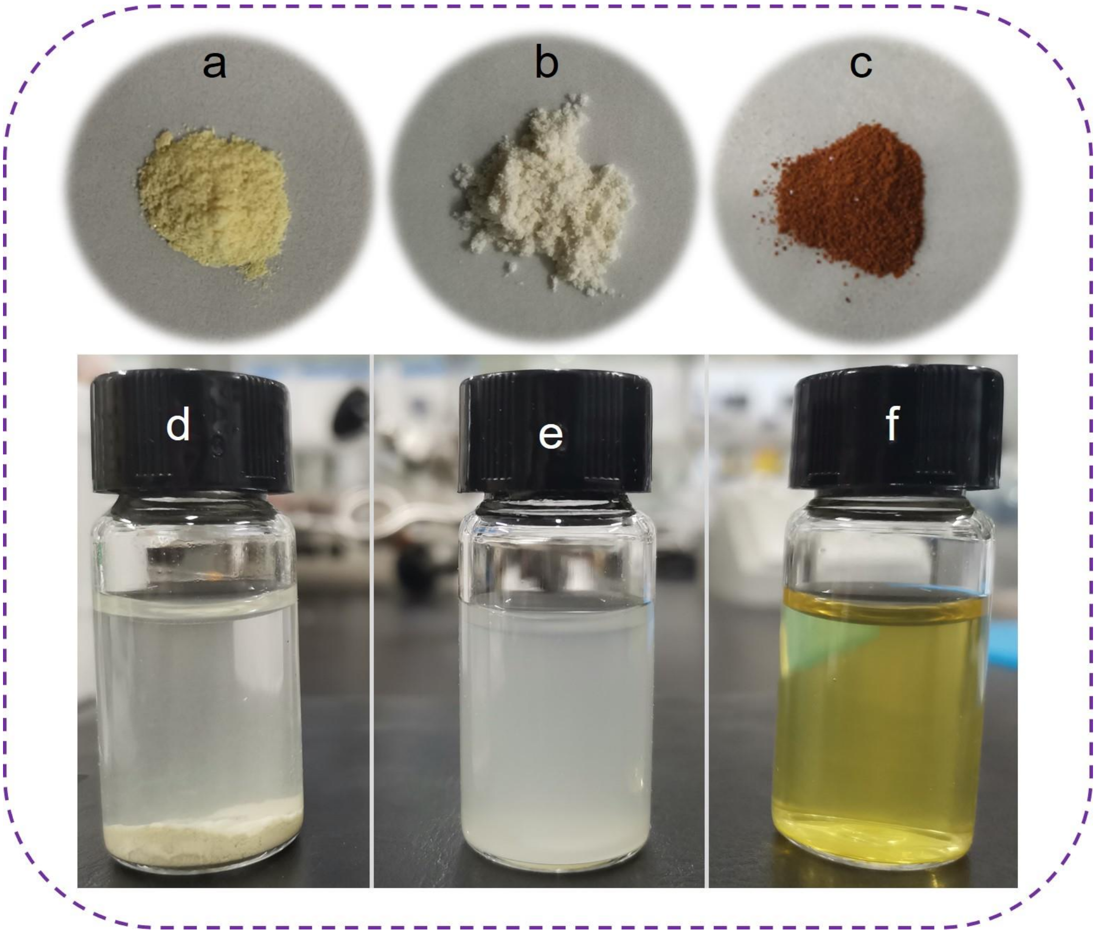

Supplementary Figure 9. Digital photograph of **a** C_3N_4 Bulk, **b** C_3N_4-OH NS, **c** C_3N_4-CN NS, **d** C_3N_4 Bulk in water after 3 days, **e** C_3N_4-OH NS in water after 3 days, **f** C_3N_4-CN NS in water after 3 days.

Compared to C_3N_4 Bulk, C_3N_4-OH NS and C_3N_4-CN NS exhibit better dispersion ability in water by virtue of their -OH and -CN groups, respectively.

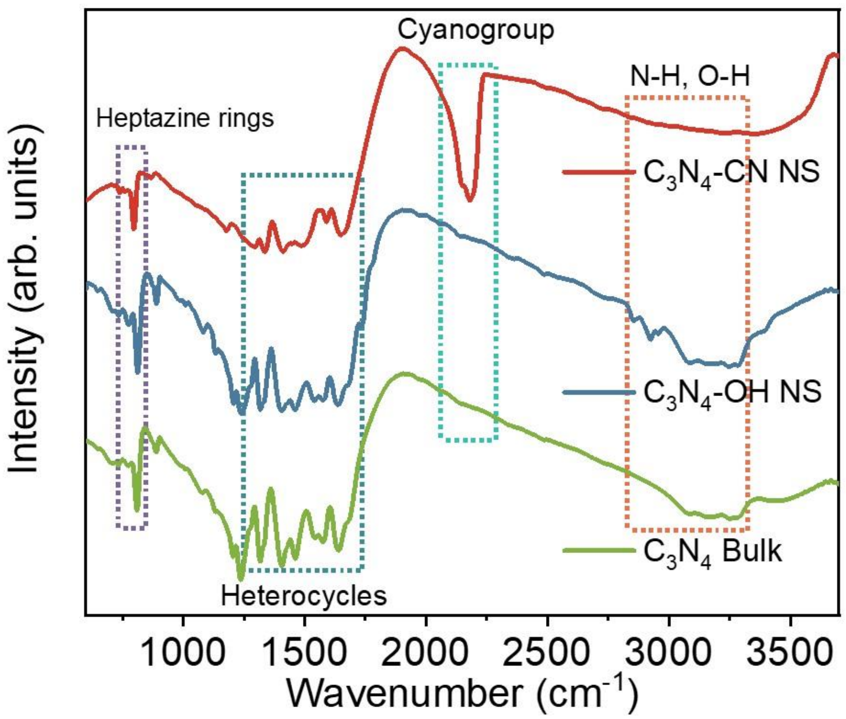

Supplementary Figure 10. FT-IR spectra of C_3N_4 Bulk, C_3N_4-OH NS and C_3N_4-CN NS.

Compared to C_3N_4 Bulk and C_3N_4-CN NS and C_3N_4-OH NS show abundant -OH and NH_2 groups due to the tailoring of NaOH. Compared to C_3N_4 Bulk and C_3N_4-OH NS, C_3N_4-CN NS show abundant -CN after the salt-assisted method.[1, 7]

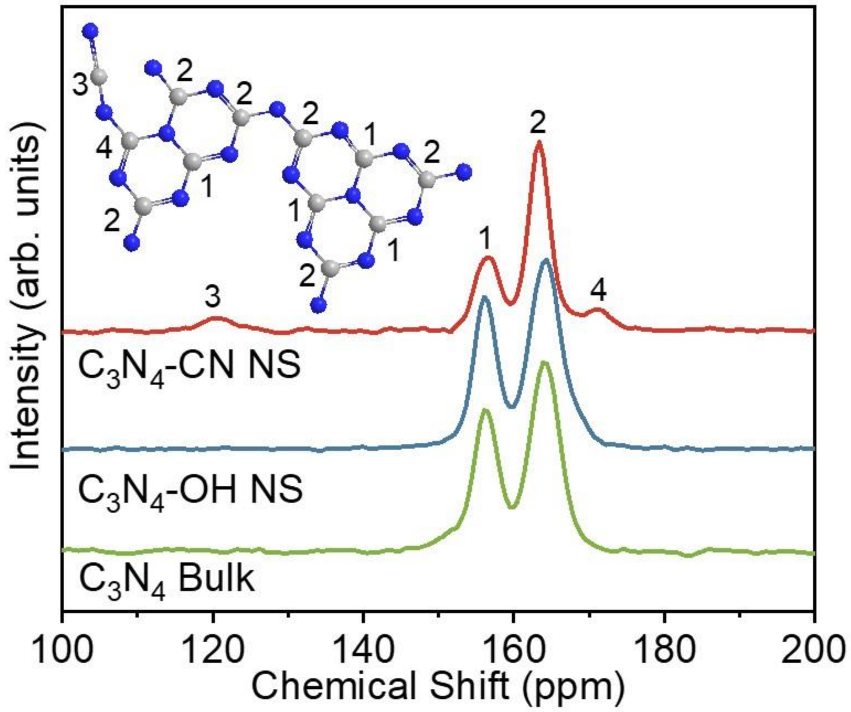

Supplementary Figure 11. Solid-state ^{13}C MAS NMR spectra of C_3N_4-CN NS, C_3N_4-OH NS and C_3N_4 Bulk.

Solid-state ^{13}C magic angle spinning (MAS) NMR measurements showed two strong peaks at 156.5 and 163.3 ppm corresponding to the chemical shifts of $C{-}N_3$ (1) and $N_2{-}C{-}NH_x$ (2) in the aromatic heterocycles, respectively.[8] Two new peaks at 120.4 and 171.0 ppm can be clearly observed for C_3N_4-CN NS, which can be ascribed to carbon atom (3) in −CN and the neighbor C atom (4), respectively.[9] For C_3N_4-CN NS, the decreased intensity of peak (1) at 156.5 ppm after destruction of partial aromatic heterocycles, leading to the appearance of −CN and the neighbor C atom (4). No new peak appears in C_3N_4-OH NS, indicating the alkaline-assisted exfoliation maintain the integrity of aromatic heterocycles.

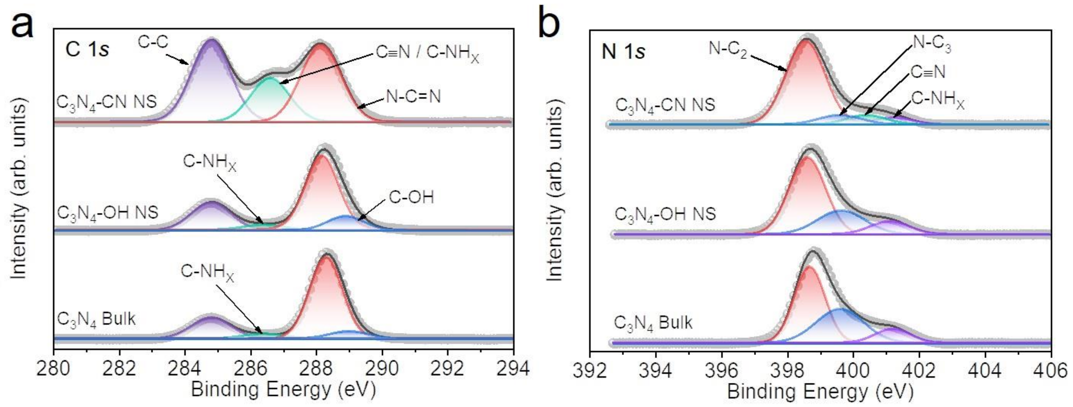

Supplementary Figure 12. High-resolution XPS spectra. a C 1*s* and **b** N 1*s* for C_3N_4 Bulk, C_3N_4-OH NS and C_3N_4-CN NS.

As is shown in C 1*s* spectra, the peaks at 284.8, 286.6, 288.2 and 288.9 eV are assigned to the adventitious carbon from the surroundings, C≡N/C−NH_x, N−C=N and C−OH, respectively.[8, 10] For N 1*s* spectra, the peaks at 398.5, 399.6, 400.4 and 401.2 eV, deconvoluted from N 1*s* can be assigned to the N atoms in two-coordinated N (C−N=C), the tri-coordinated N (N−$(C)_3$), the −CN and −NH_x, respectively.[8, 10] Compared to C_3N_4 Bulk, C_3N_4-OH NS exhibit a higher percentage of C−OH in C 1*s* and a lower percentage of N−C_3 in N 1s due to the tailoring of NaOH. Both C and N 1*s* show C_3N_4-CN NS contain abundant -CN.

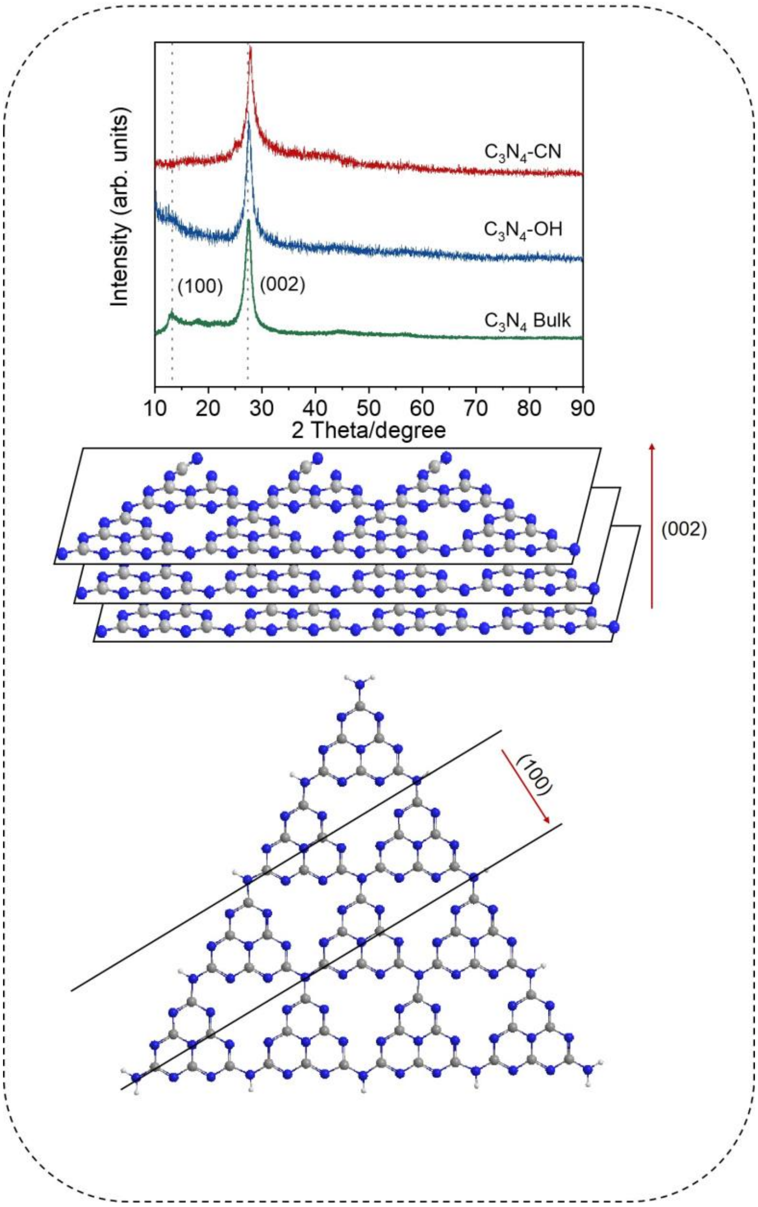

Supplementary Figure 13. XRD patterns of C_3N_4 Bulk, C_3N_4-OH NS and C_3N_4-CN NS.

Compared to C_3N_4 Bulk, the peak of (002) plane for C_3N_4-CN and C_3N_4-OH NS slightly and positively shift by ~0.43° and ~0.35°, respectively, indicating the expansion of the periodic interlayer due to the amorphization and K^+(Na^+) intercalation during the preparation.[11] The disappeared peak of (100) demonstrate that the periodicity of C_3N_4 is drastically weakened through the introduction of terminal groups, -CN and -OH.

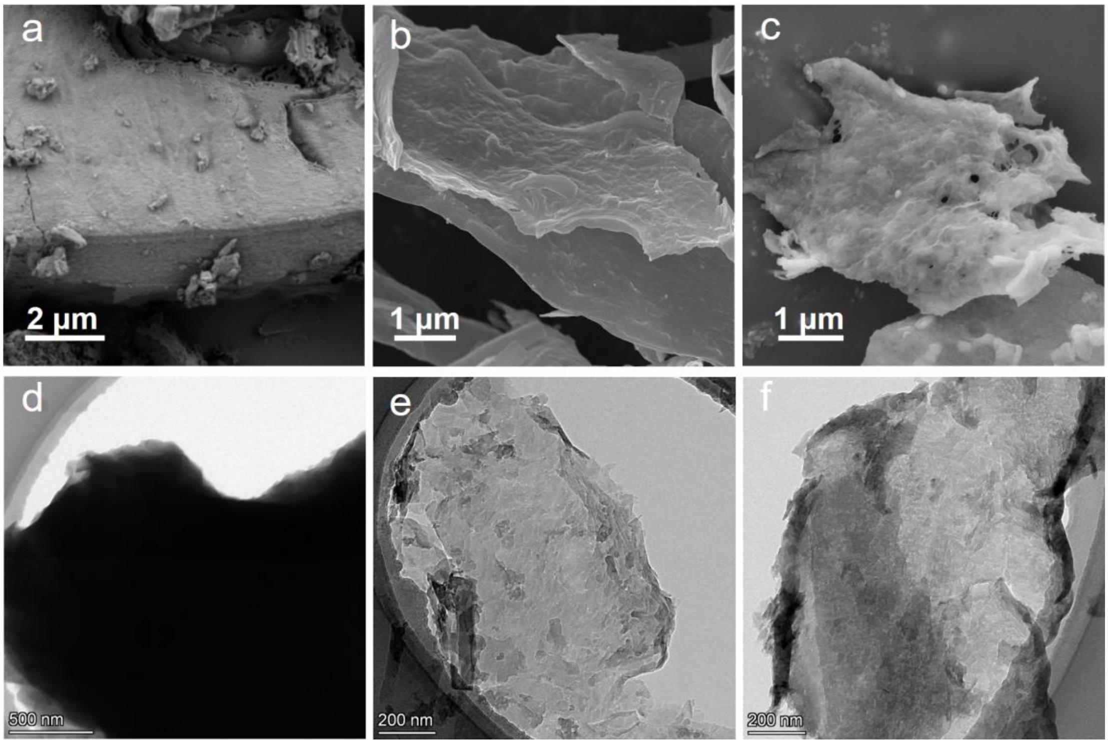

Supplementary Figure 14. SEM images of **a** C_3N_4 Bulk, **b** C_3N_4-CN NS, **c** C_3N_4-OH NS; TEM images of **d** C_3N_4 Bulk, **e** C_3N_4-CN NS, **f** C_3N_4-OH NS.

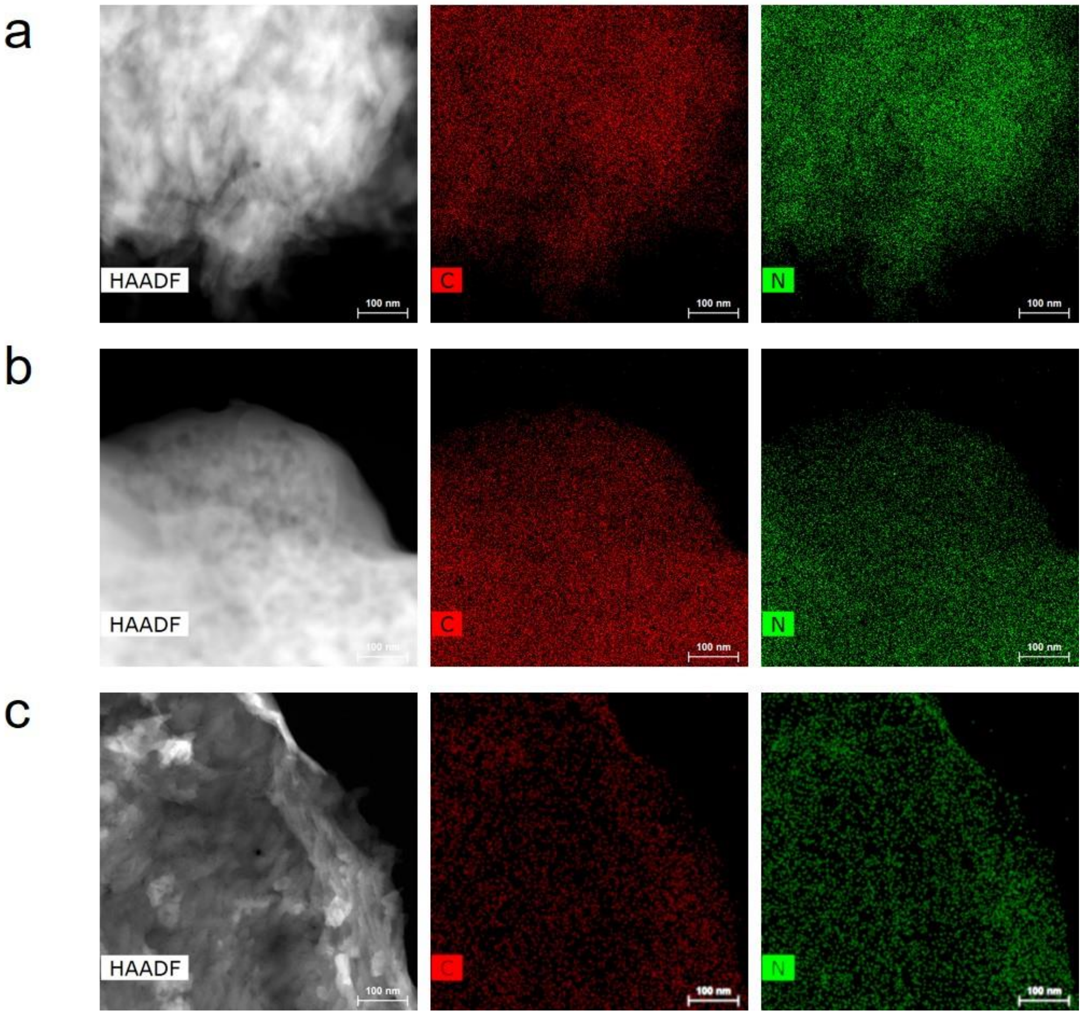

Supplementary Figure 15. EDS mapping image of **a** C_3N_4 Bulk, **b** C_3N_4-CN NS, **c** C_3N_4-OH NS.

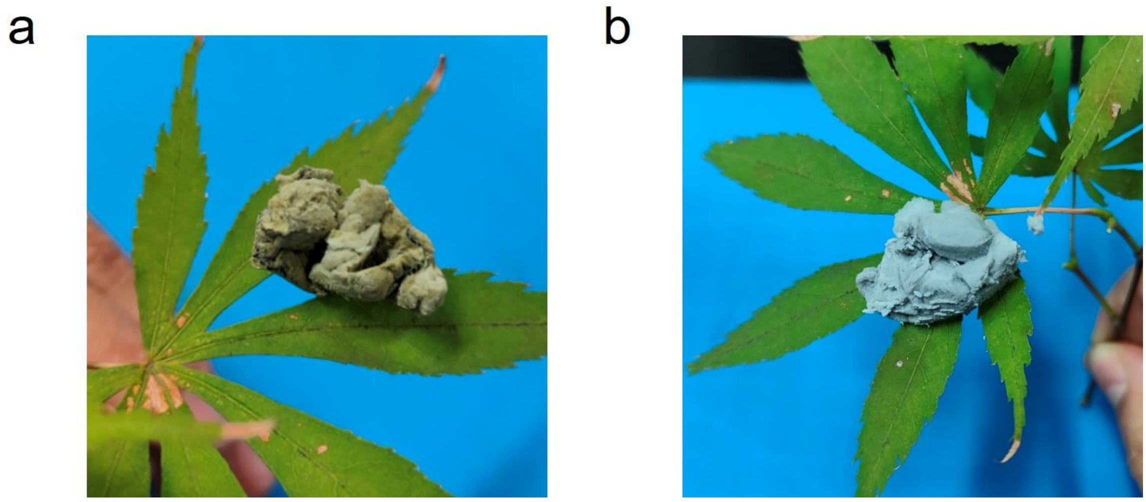

Supplementary Figure 16. Digital photograph of aerogels. a The precursor of $Ni@C_3N_4$-CN after freeze drying. **b** The precursor of $Ni@C_3N_4$ after freeze drying.

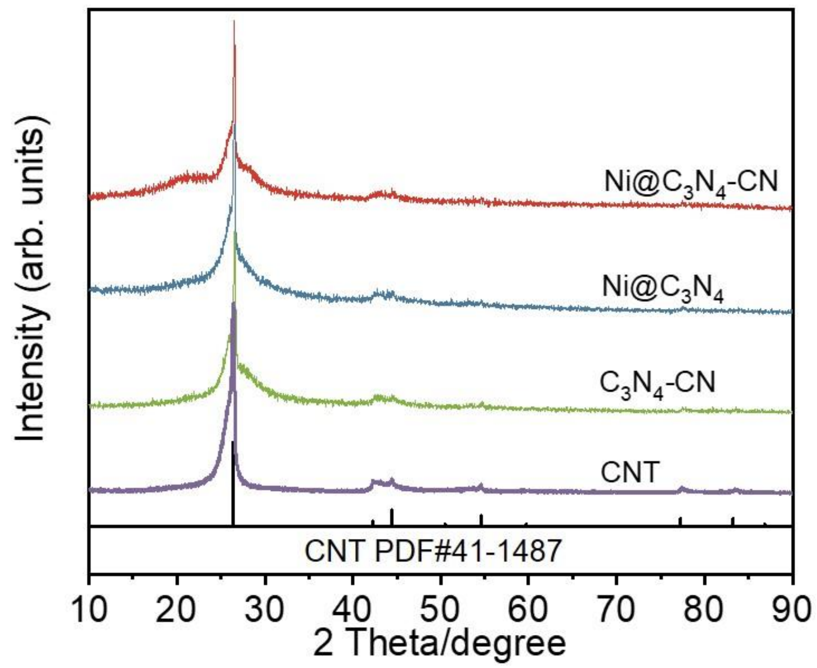

Supplementary Figure 17. XRD patterns of Ni@C_3N_4-CN, Ni@C_3N_4, C_3N_4-CN catalyst and CNT.

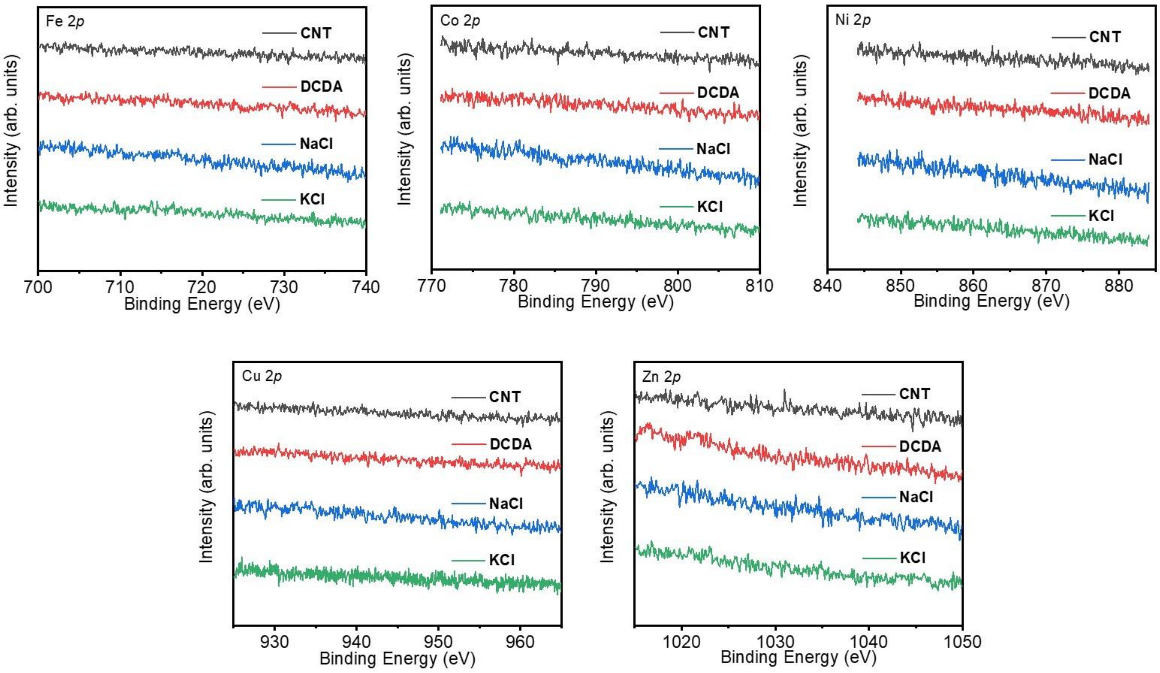

Supplementary Figure 18. High-resolution XPS spectra of Fe 2*p*, Co 2*p*, Ni 2*p*, Cu 2*p*, and Zn 2*p* for the raw materials.

As shown in Supplementary Figure S10, no XPS signal of Fe 2*p*, Ni 2*p*, Co 2*p*, Cu 2*p* and Zn 2*p* is detected in all the precursor, indicating that the metal contamination is neglected.

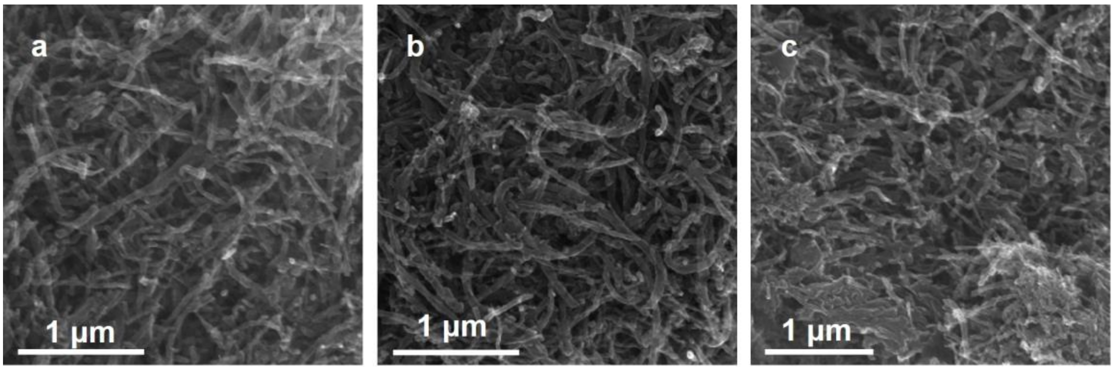

Supplementary Figure 19. SEM images of **a** Ni@C_3N_4-CN, **b** Ni@C_3N_4 and **c** C_3N_4-CN catalyst.

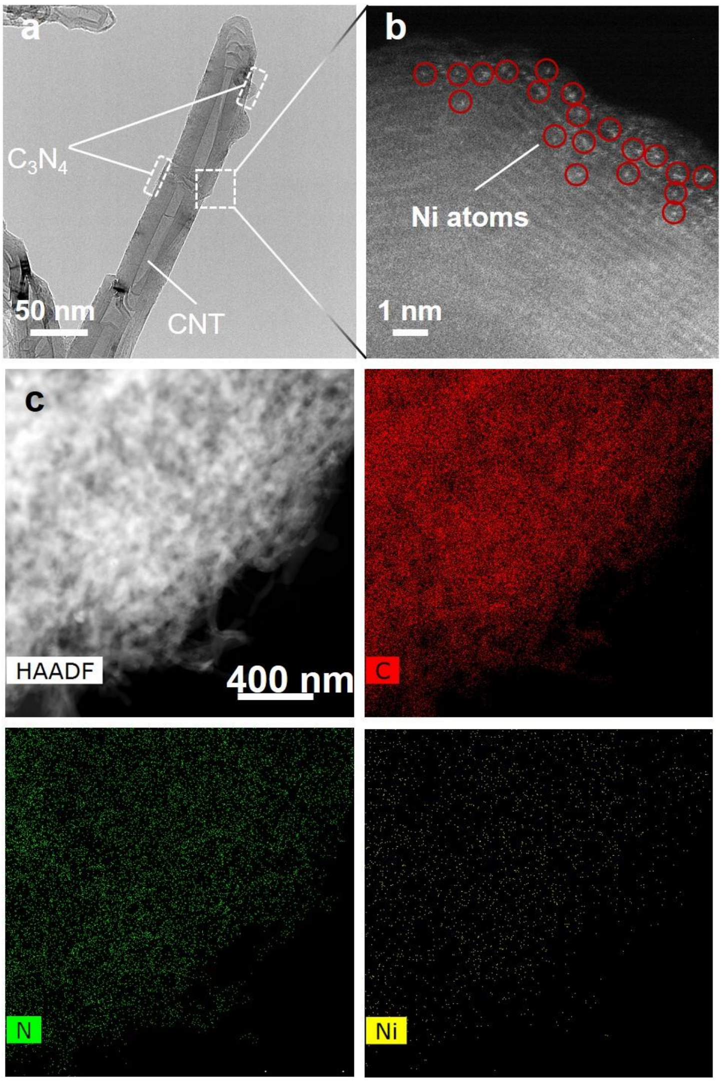

Supplementary Figure 20. a TEM image of Ni@C_3N_4. **b** AC HAADF-STEM image of Ni@C_3N_4. **c** EDS mapping image of Ni@C_3N_4.

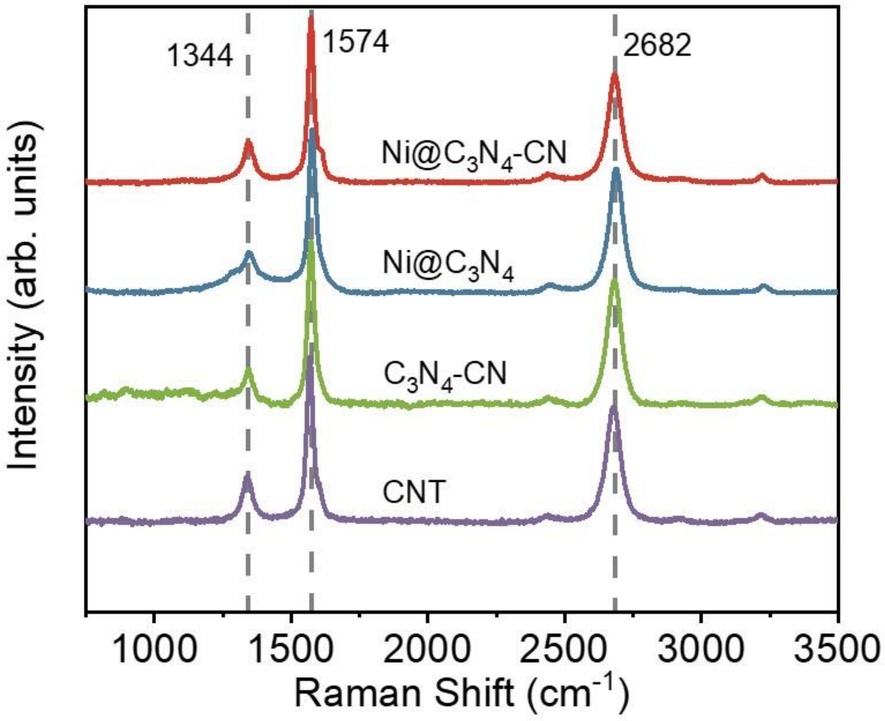

Supplementary Figure 21. Raman spectra of Ni@C_3N_4-CN, Ni@C_3N_4, C_3N_4-CN catalyst and CNT.

The peaks at 1344, 1574 and 2682 cm^{-1} are attributed to the *D* band, *G* band and *G′* band of CNT, respectively[12].

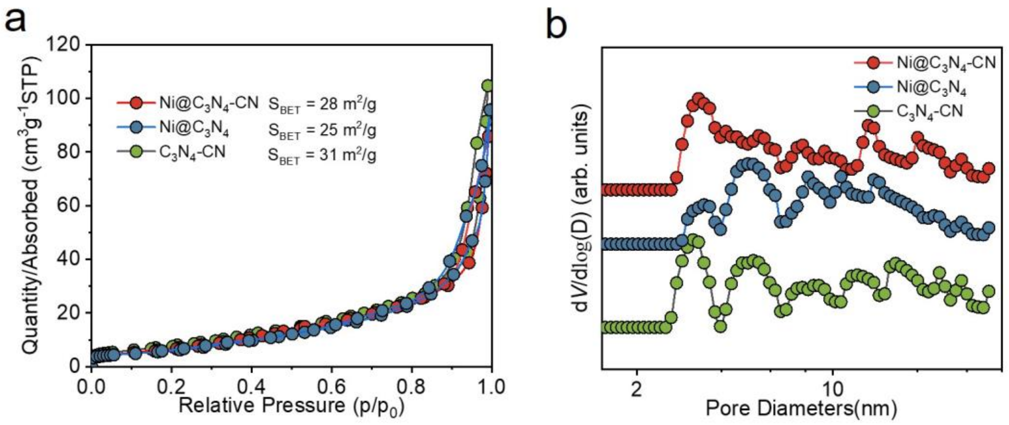

Supplementary Figure 22. a Nitrogen sorption isotherm for different catalysts. **b** Pore size distribution of different catalysts.

Ni@C_3N_4-CN, Ni@C_3N_4 and C_3N_4-CN catalyst show high similarity in specific surface and pore size distribution.

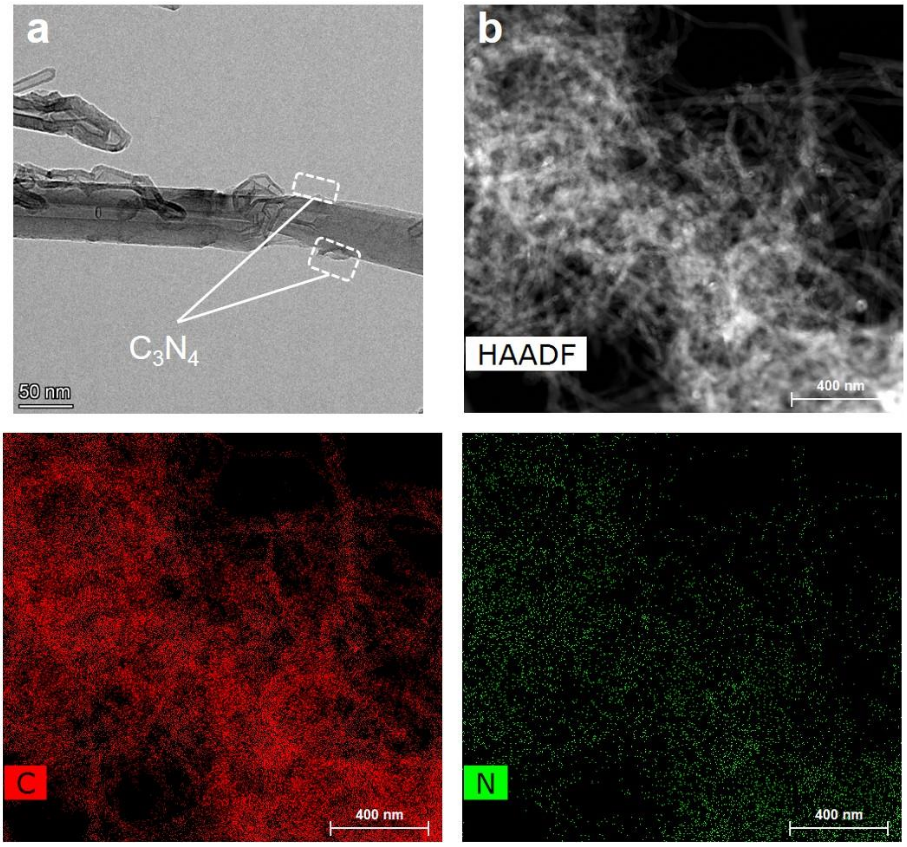

Supplementary Figure 23. a TEM image of C_3N_4-CN catalyst. **b** EDS mapping image of C_3N_4-CN catalyst.

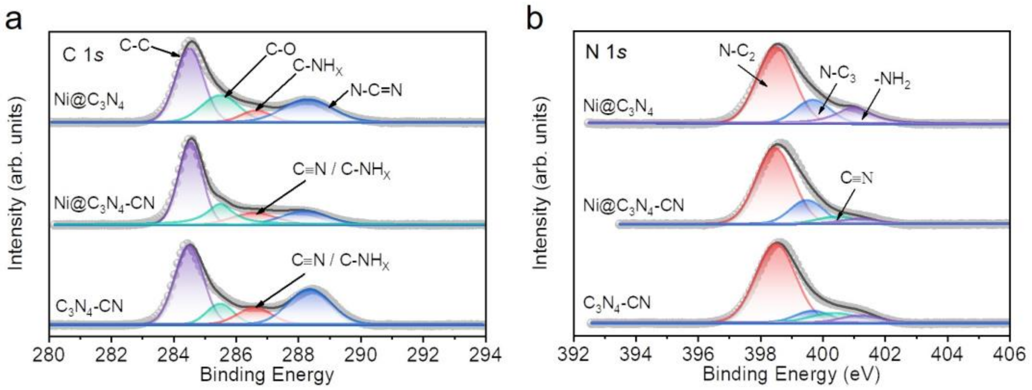

Supplementary Figure 24. High-resolution XPS spectra of **a** C 1*s* and **b** N 1*s* for Ni@C_3N_4-CN, Ni@C_3N_4, C_3N_4-CN catalyst.

As is shown in C 1*s* spectra, the peaks at 284.8, 286.6, 288.2 and 288.9 eV are attributed to the adventitious carbon from the surroundings, C−O, C≡N/C−NH_x and N−C=N, respectively.[8, 10] Four peaks at about 401.2, 400.4, 399.6 and 398.5 eV, deconvoluted from N 1*s* can be assigned to the N atoms in the surface amino groups, the −CN, the tri-coordinated N (N−$(C)_3$) and two-coordinated N (C−N=C), respectively. N 1*s* of C_3N_4-CN has changed obviously after the addition of Ni sites, indicating Ni sites coordinate with N atoms of C_3N_4.

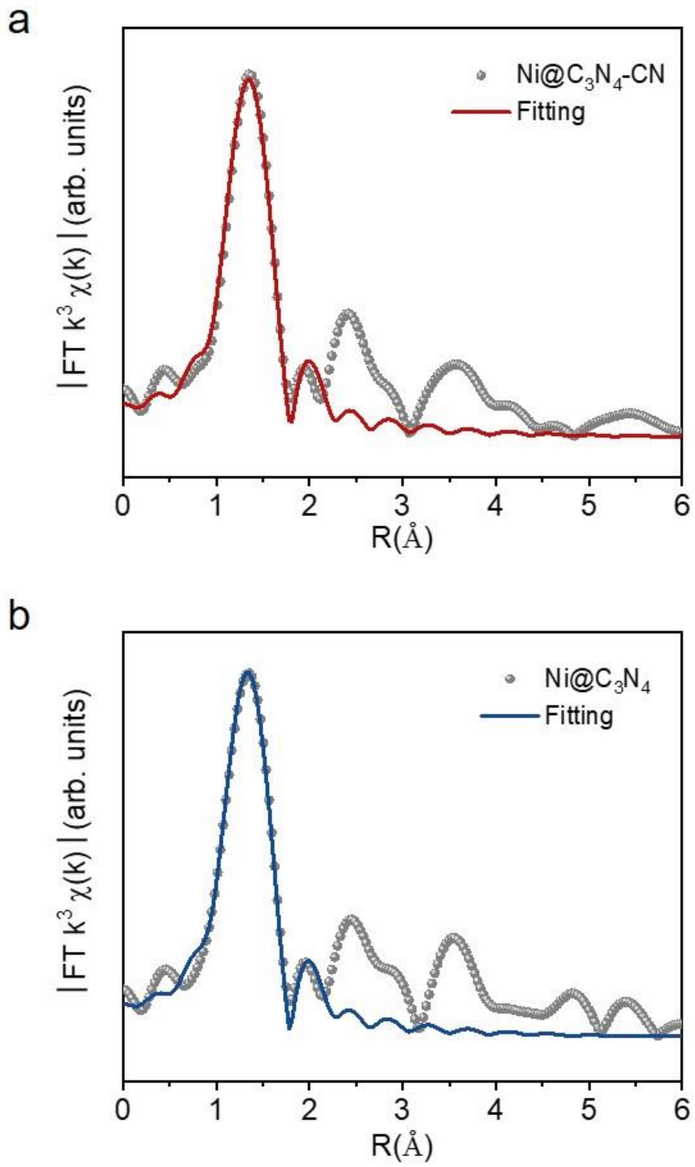

Supplementary Figure 25. EXAFS fitting for Ni@C_3N_4-CN and Ni@C_3N_4.

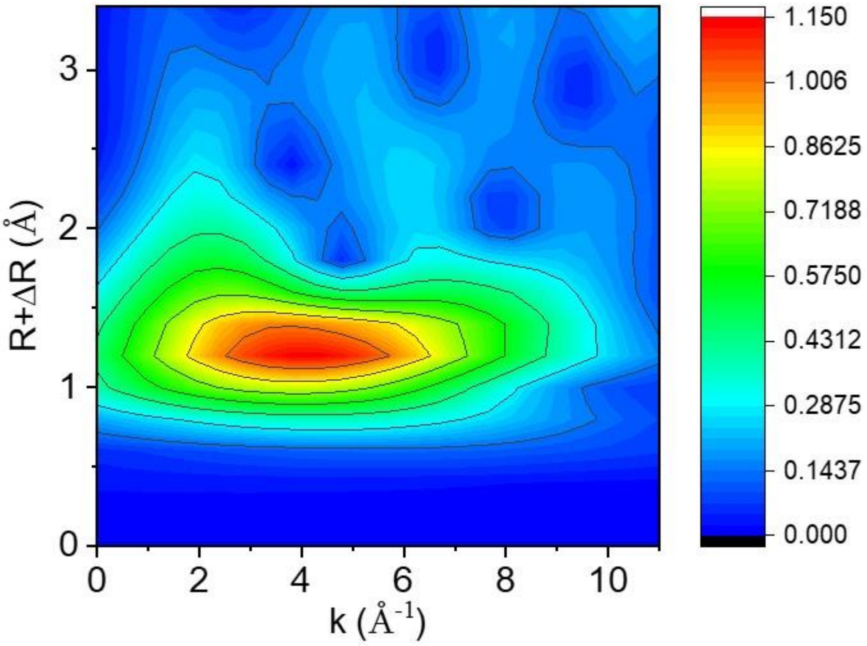

Supplementary Figure 26. WT-EXAFS plot for Ni@C_3N_4.

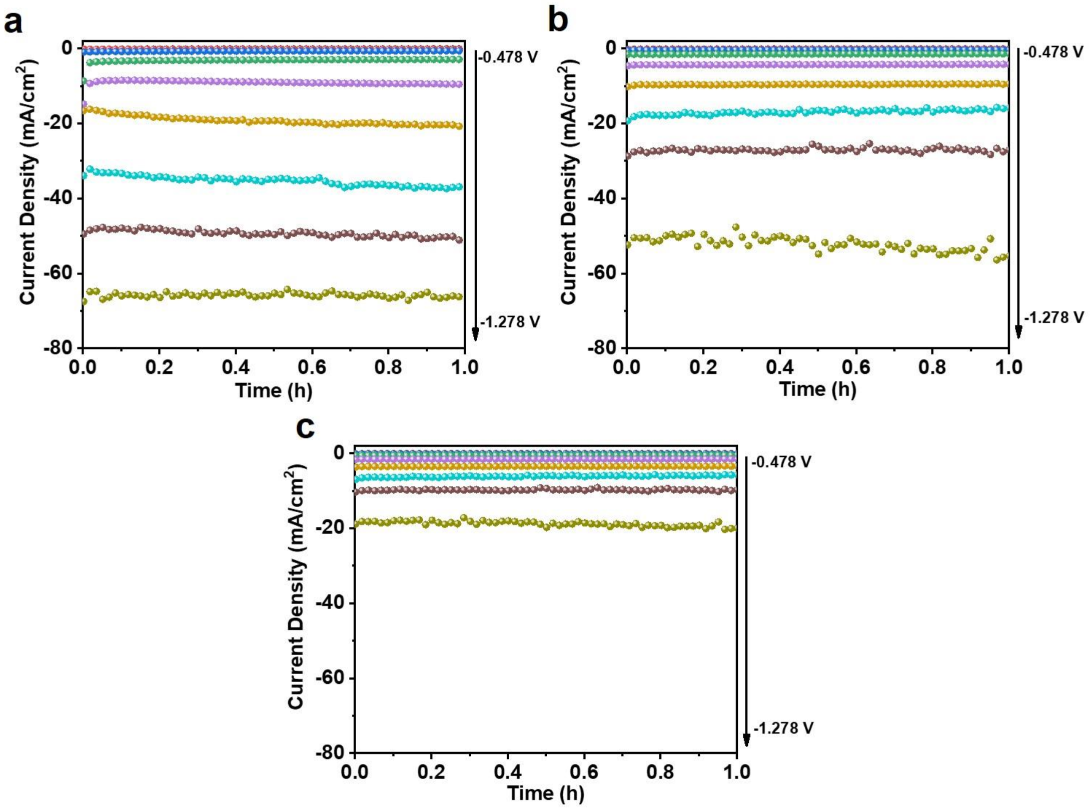

Supplementary Figure 27. i-t curves of catalysts from -0.478 V to −1.278 V vs. RHE in CO_2-saturated 0.5 M $KHCO_3$ solution. a Ni@C_3N_4-CN. **b** Ni@C_3N_4. **c** C_3N_4-CN catalyst.

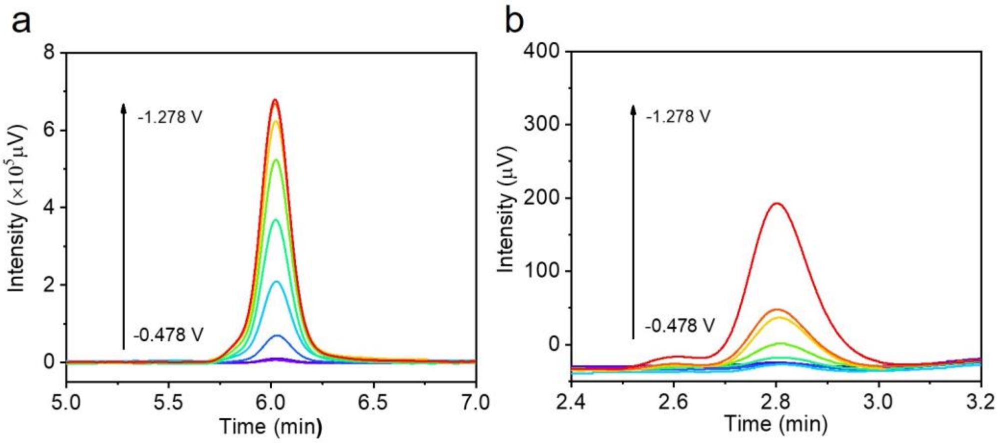

Supplementary Figure 28. The spectra of Gas chromatograph (GC) for **a** CO and **b** H_2 produced by Ni@C_3N_4-CN.

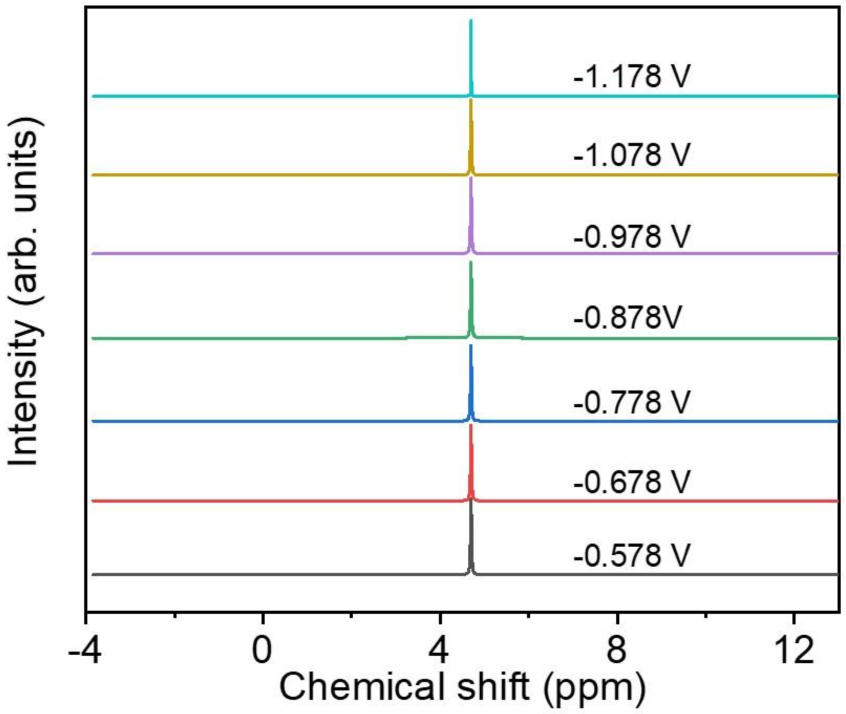

Supplementary Figure 29. ^{1}HNMR spectra for Ni@C_3N_4-CN after CO_2 reduction electrolysis at different potential in CO_2 saturated 0.5 M $KHCO_3$ solution. No C_2 and liquid products (such as ethanol, methanol) was observed by ^{1}H NMR.

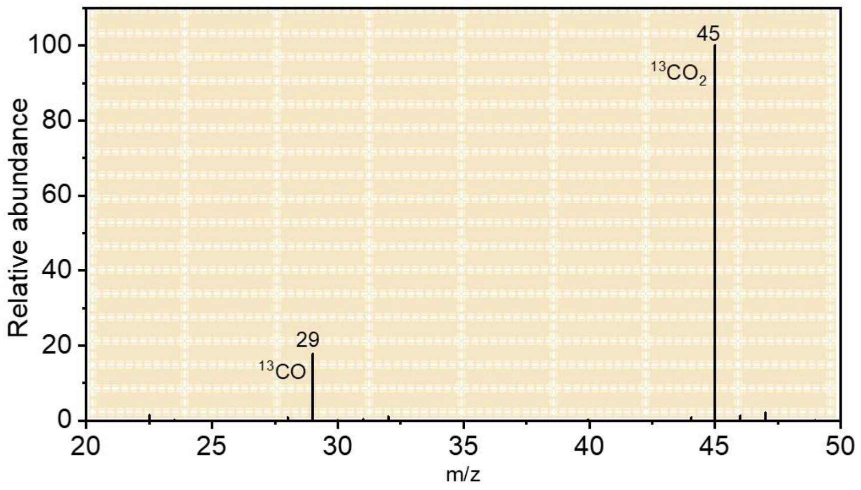

Supplementary Figure 30. Mass spectrometry signal of CO_2RR using $^{13}CO_2$ as the feedstock.

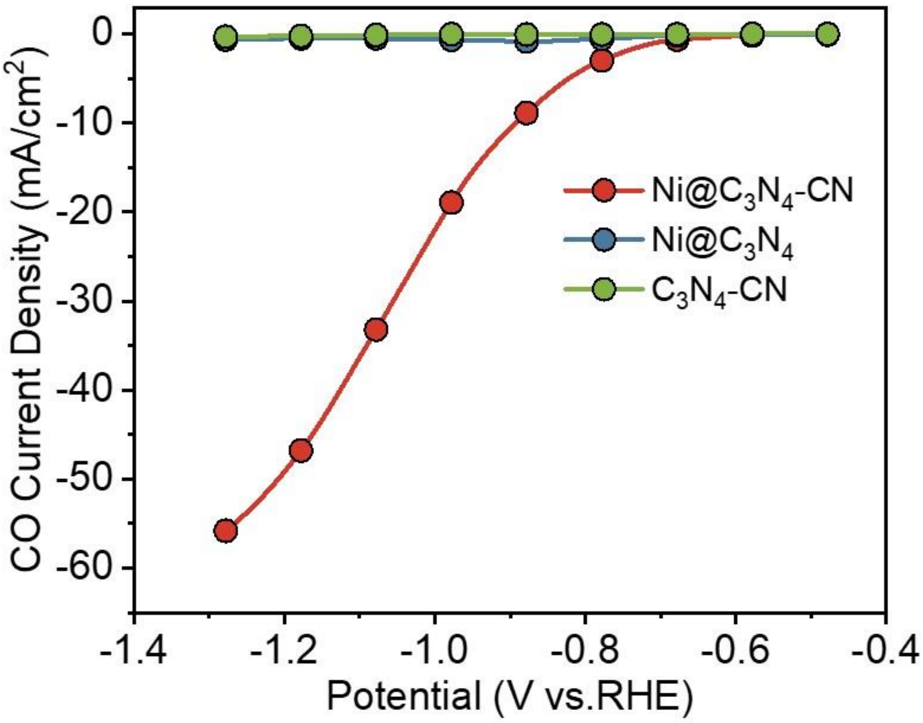

Supplementary Figure 31. CO partial current density over different catalysts in pure CO_2 saturated 0.5 M $KHCO_3$ solution.

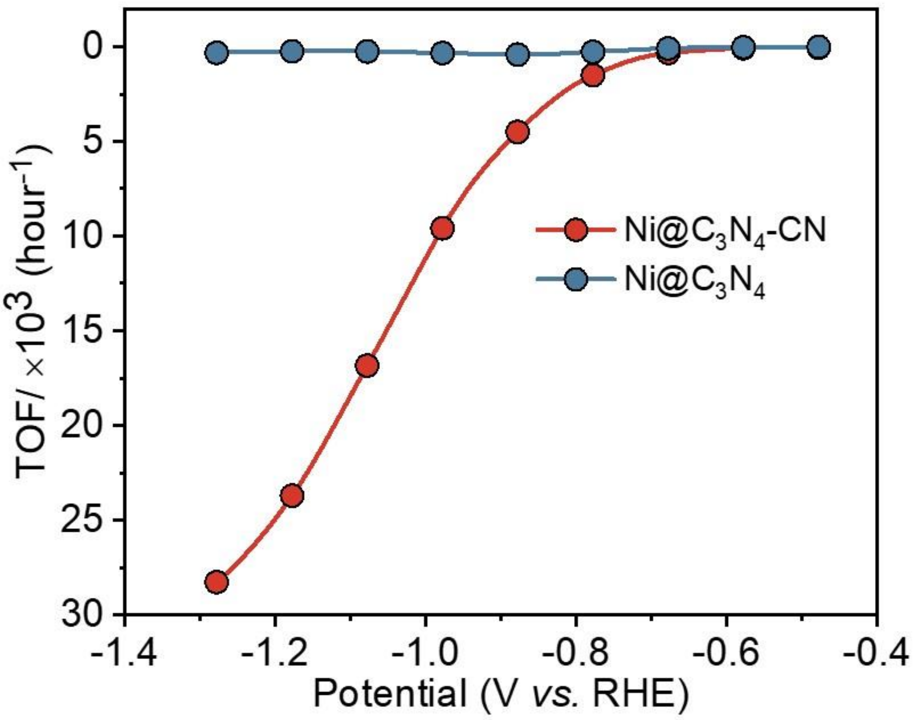

Supplementary Figure 32. TOF of CO production for different catalysts in CO_2-saturated 0.5 M $KHCO_3$ solution in pure CO_2 saturated 0.5 M $KHCO_3$ solution.

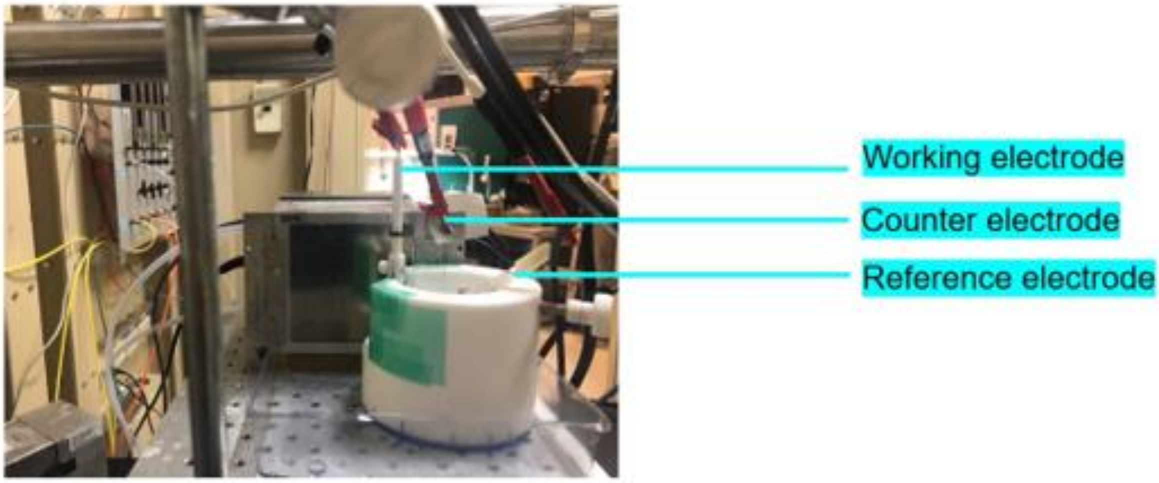

Supplementary Figure 33. Digital photograph of in situ XAS equipment.

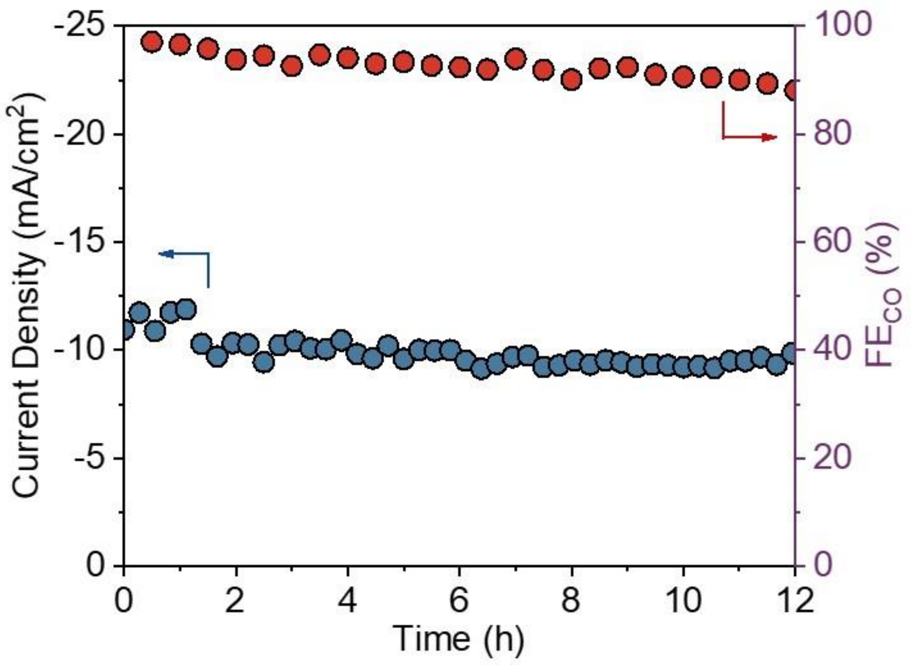

Supplementary Figure 34. Stability of Ni@C_3N_4-CN at a potential of −0.878 V vs. RHE in H-cell in pure CO_2 saturated 0.5 M $KHCO_3$ solution.

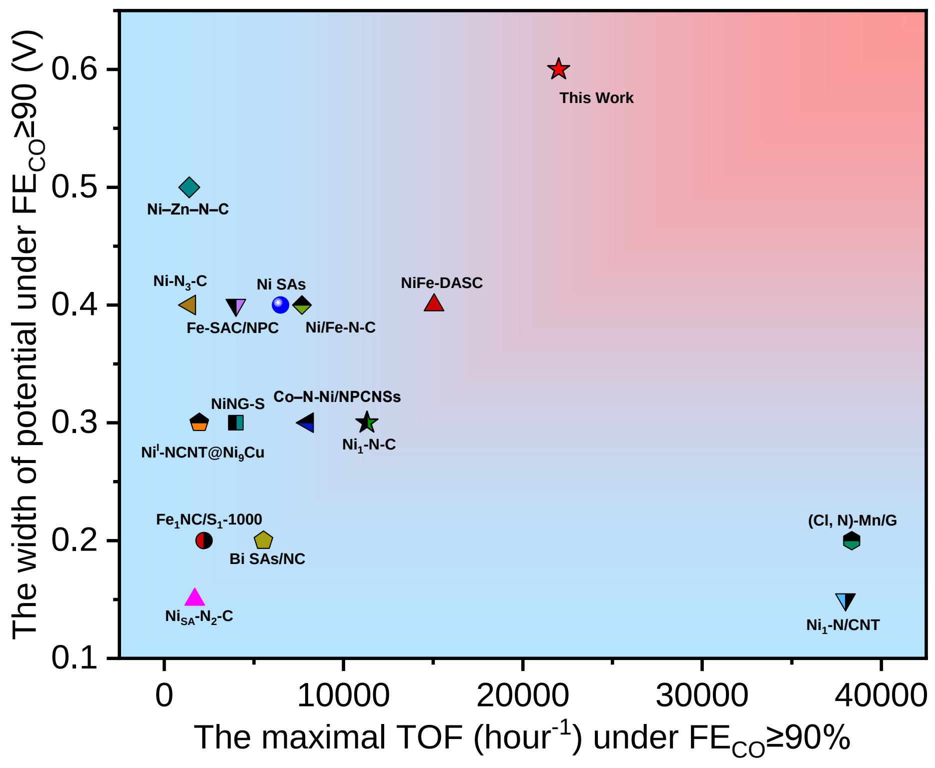

Supplementary Figure 35. Performance comparison of catalysts with various metal sites for CO_2RR to CO in H-cell with $KHCO_3$ electrolyte.[3-4, 13]

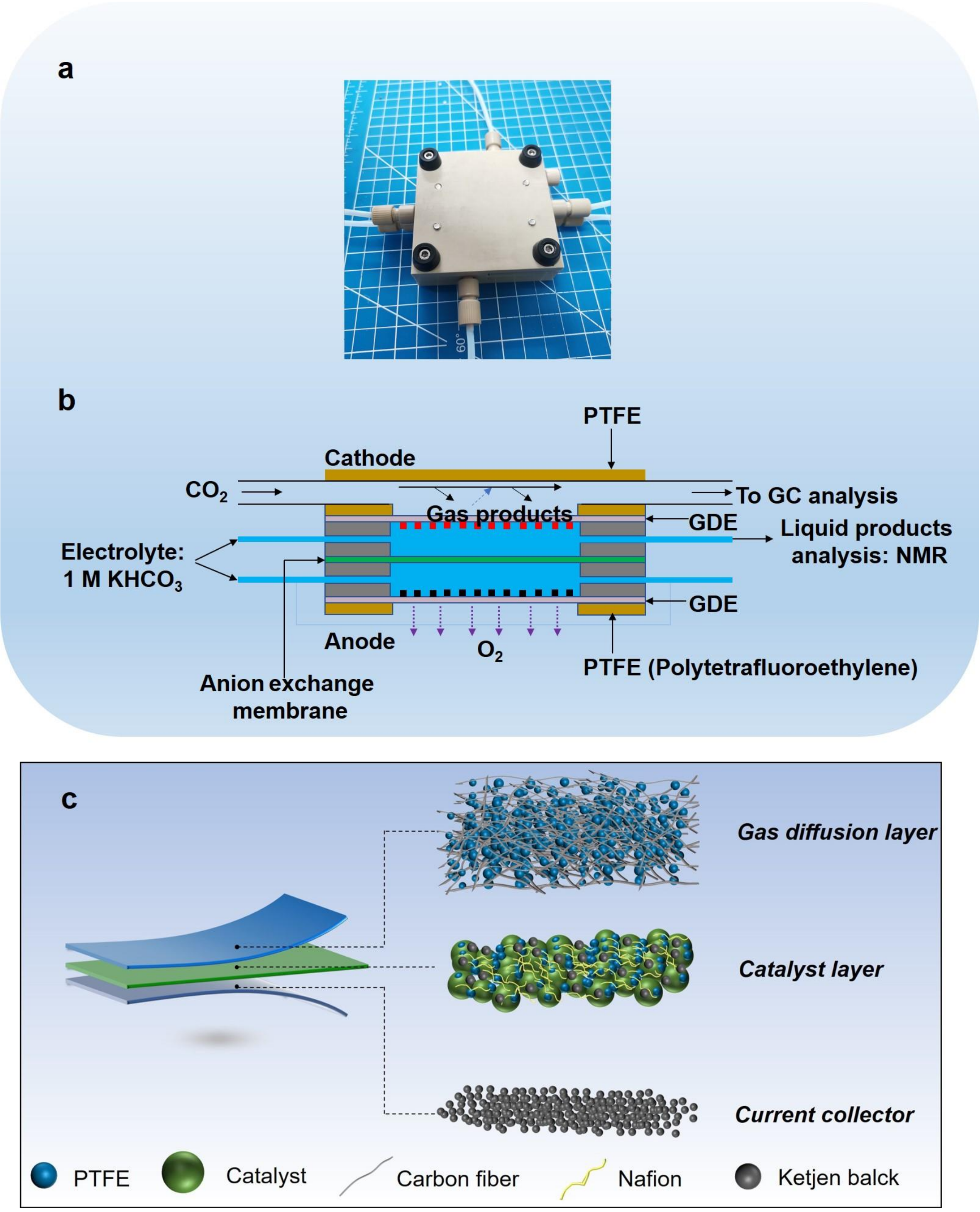

Supplementary Figure 36. a Digital photograph of flow cell. **b** Schematic representation of the flow cell. **c** Schematic representation of electrode on flow cell.

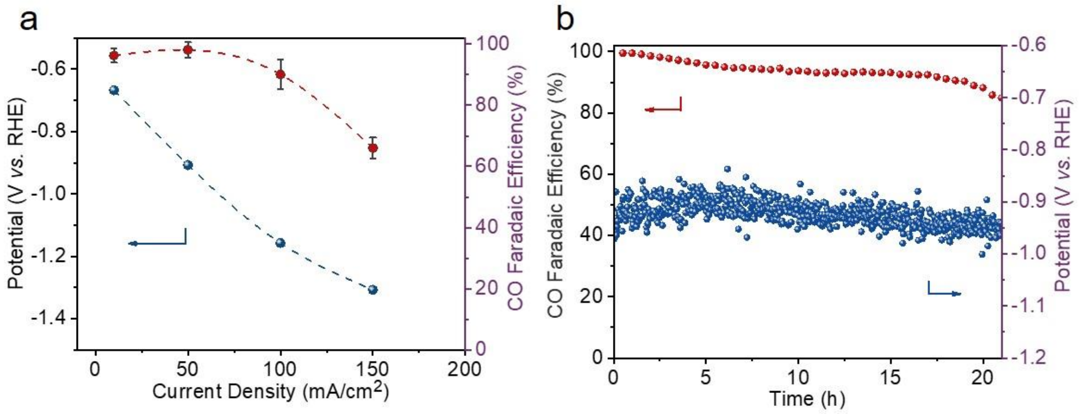

Supplementary Figure 37. a Potentials and FE_{CO} at different current densities of Ni@C_3N_4-CN in flow cell under 30% CO_2 concentration. **b** Stability of Ni@C_3N_4-CN at a current density of 50 mA/cm^2 in flow cell under 30% CO_2 concentration. The error bars correspond to the standard deviations of measurements over three separately prepared samples under the same testing conditions.

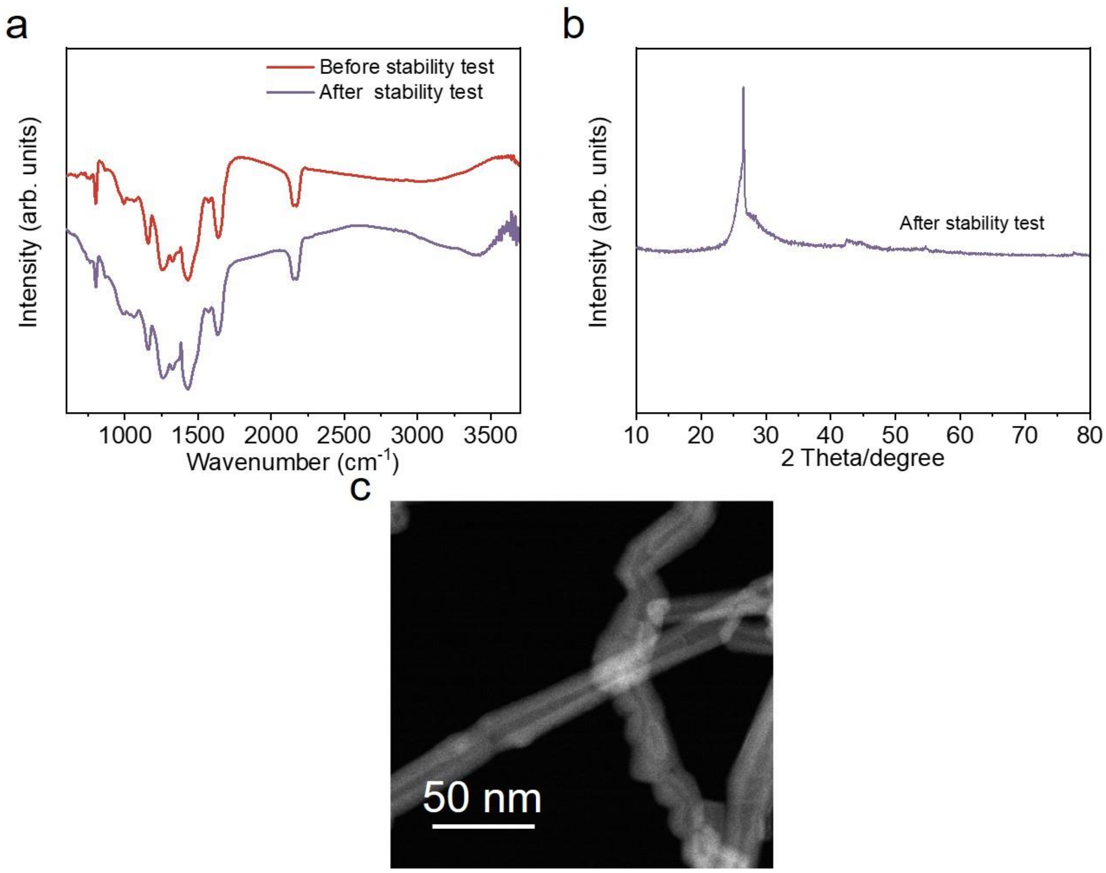

Supplementary Figure 38. a FT-IR spectra of Ni@C_3N_4-CN before and after stability test. **b** XRD patterns Ni@C_3N_4-CN after stability test. **c** AC HAADF-STEM image of Ni@C_3N_4-CN after stability test.

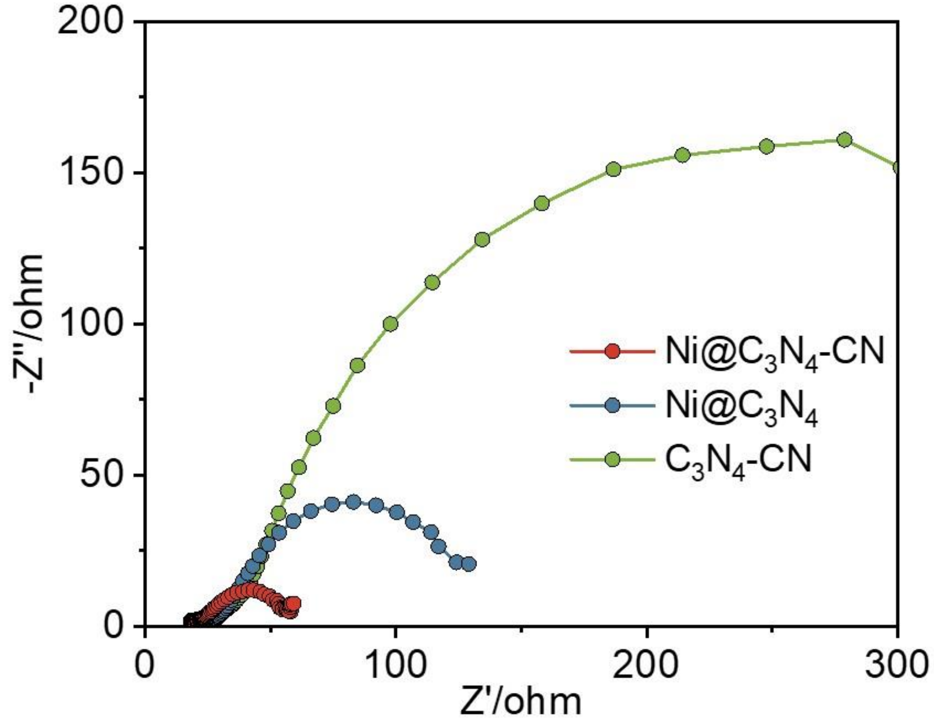

Supplementary Figure 39. Nyquist plots in CO_2-saturated 0.5 M $KHCO_3$ solution for different catalysts.

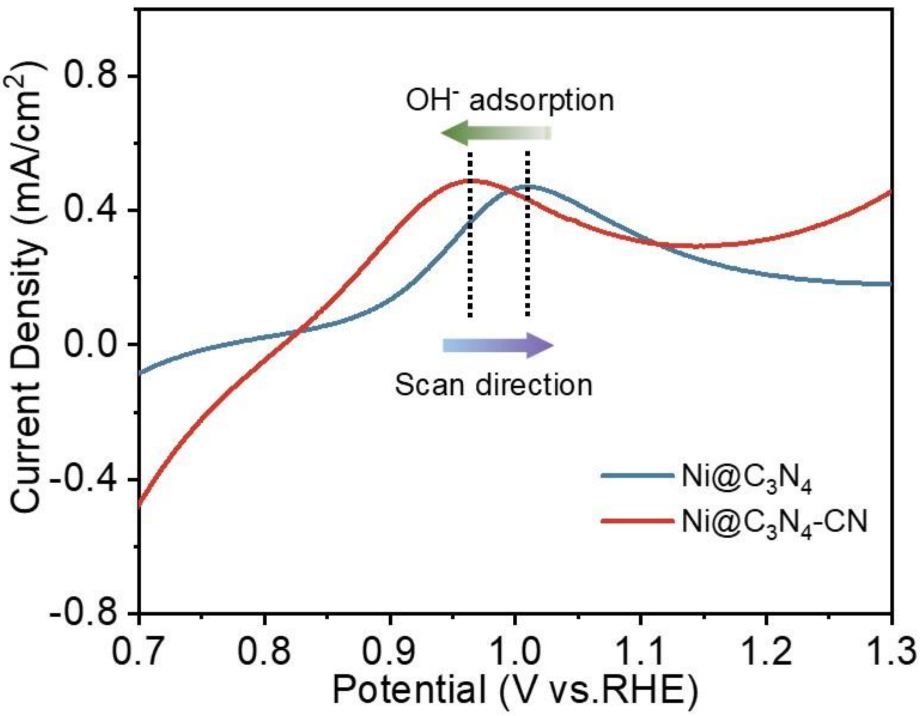

Supplementary Figure 40. Electrochemical activation test.

The oxidative LSV scans was conducted in N_2-satured 0.5 M KOH with scan rate of 50 mV/s. The dissociative $*CO_2^-$ is unstable in aqueous solution, because it is very difficult to detect the image of $*CO_2^-$ directly through simple electrochemical measurement. For evaluating the ability of CO_2 adsorption, we employed OH^- as a surrogate to evaluate the binding affinity of $*CO_2^-$, in which the stronger OH^- adsorption, the stronger $*CO_2^-$ adsorption. We can obtain the ability of OH^- adsorption through the oxidative LSV scans in N_2-saturated 0.5 M NaOH electrolyte.[14]

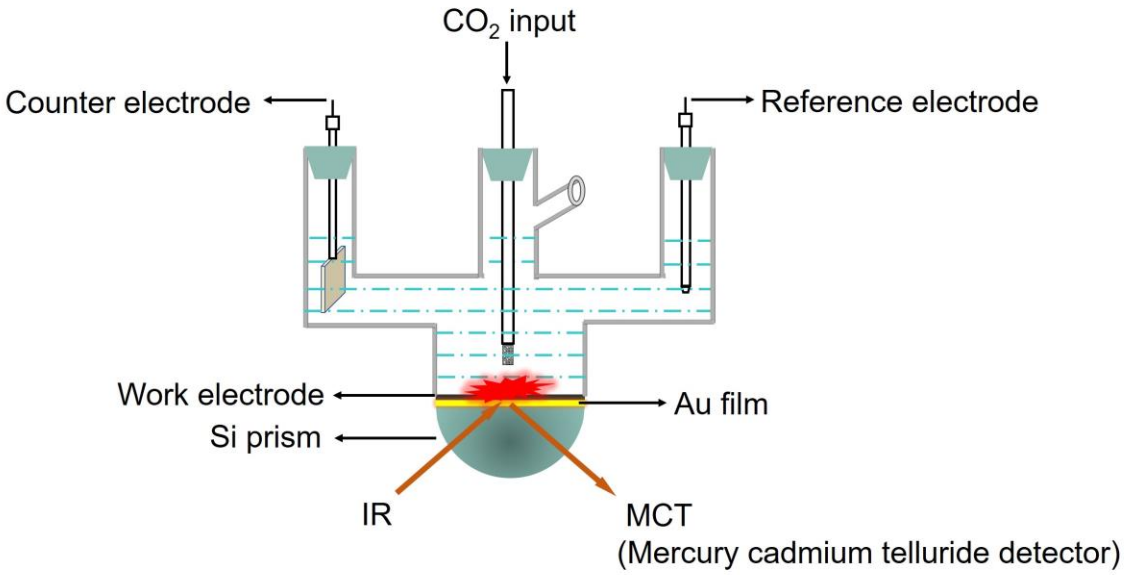

Supplementary Figure 41. Schematic representation of attenuated total reflection surface-enhanced infrared absorption spectroscopy (ATR-SEIRAS).

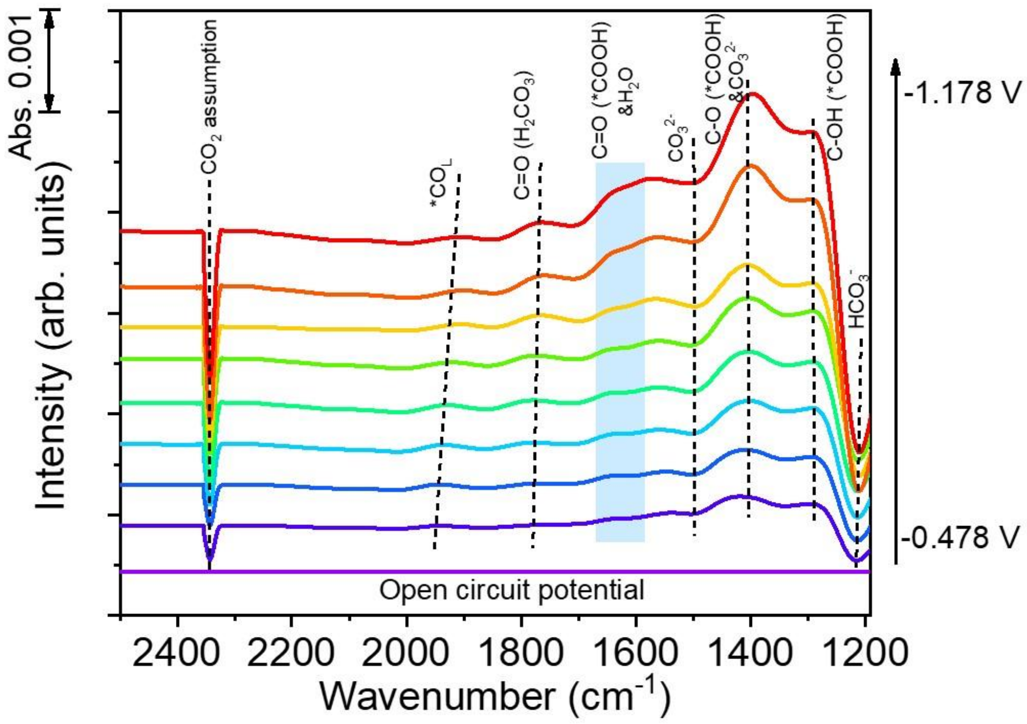

Supplementary Figure 42. In situ ATR-IR spectra of Ni@C_3N_4.

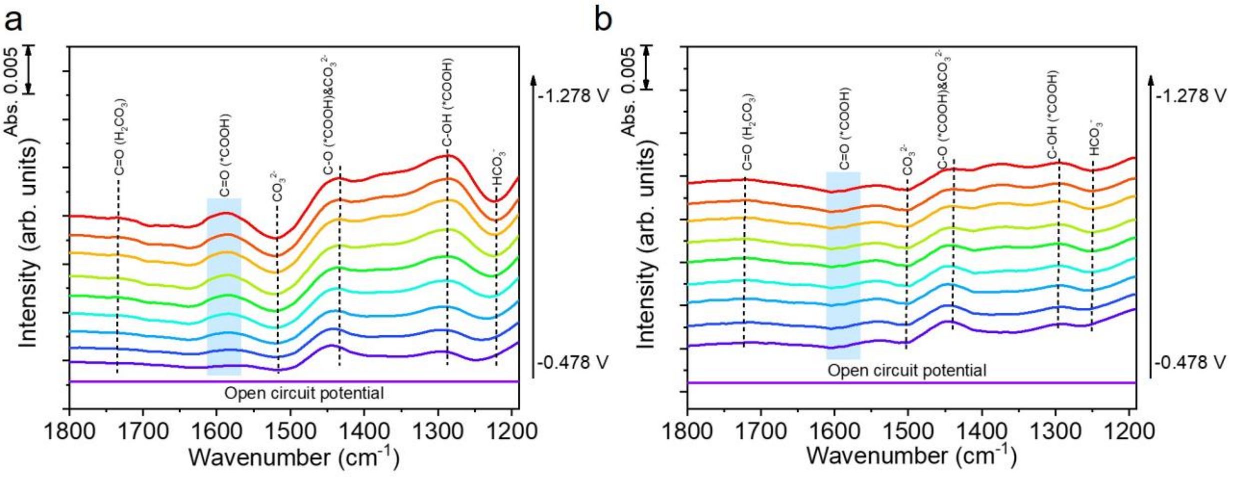

Supplementary Figure 43. a In situ ATR-IR spectra of Ni@C_3N_4-CN in D_2O electrolyte. **b** In situ ATR-IR spectra of Ni@C_3N_4 in D_2O electrolyte.

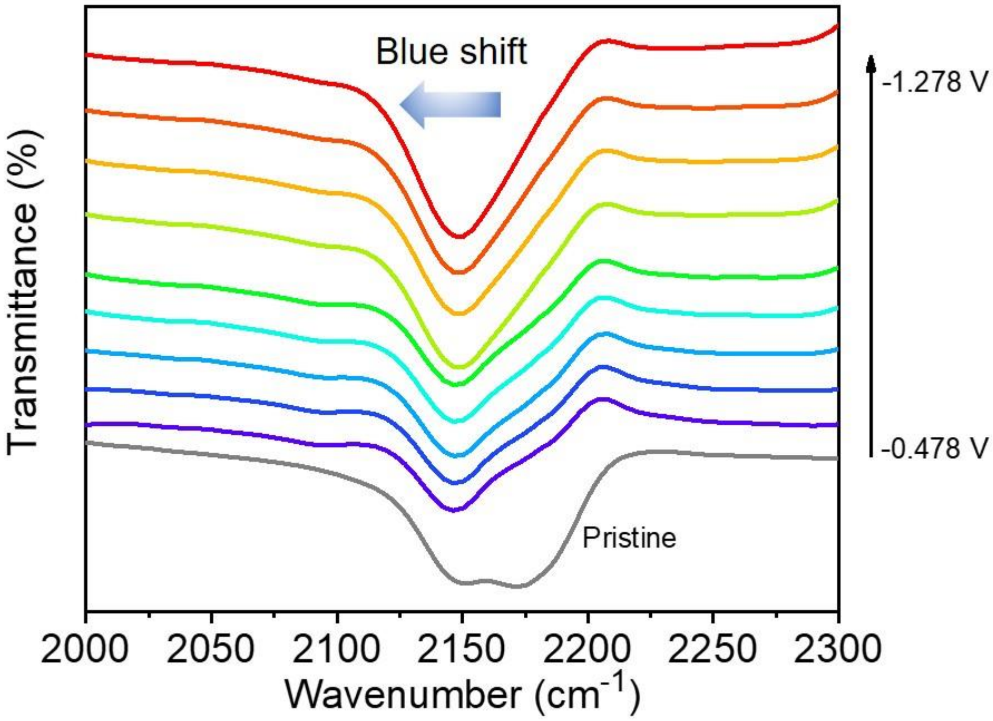

Supplementary Figure 44. Total FT-IR spectra of −CN for Ni@C_3N_4-CN under different potential.

The total FT-IR spectra of −CN was obtained from the pristine value and variation in based on the following equation：

$$A=\log(\frac{1}{T}) \tag{1}$$

A: Absorbance

T: Transmittance (%)

Supplementary Table 1. EXAFS data fitting results of Ni@C_3N_4-CN and Ni@C_3N_4.

**Sample**	**Shell**	**CN**	**R(Å)**	**σ^2**	**$\triangle E_0$(eV)**	**S_0^2**	**R factor**
Ni@C_3N_4-CN	Ni-N	3.7	1.83±0.01	0.008	3.68±2.75	0.76	0.007
Ni@C_3N_4	Ni-N	3.8	1.82±0.01	0.009	3.81±2.87	0.76	0.007

Supplementary Table 2. The energy efficiency (EE) of CO_2 to CO for Ni@C_3N_4-CN in flow-cell under both saturated and dilute CO_2 (30%) conditions.

**Flow cell (100% CO_2)**			**Flow cell (30% CO_2)**		
**E (V)**	**FE (%)**	**EE (%)**	**E (V)**	**FE (%)**	**EE (%)**
-0.61	96.8	70.4	-0.67	96.3	68.0
-0.78	96.2	64.0	-0.91	98.1	61.5
-0.82	93.8	61.1	-1.16	90.1	50.6
-0.88	92.3	58.7	-1.31	66.0	34.9

Supplementary Table 3. The single pass efficiency (SPC) of CO_2 to CO for Ni@C_3N_4-CN in flow-cell under both saturated and dilute CO_2 (30%) conditions.

**Flow cell (100% CO_2)**			**Flow cell (30% CO_2)**		
j (mA)	**flow rate (L/min)**	**SPCC (%)**	***j (mA)***	**flow rate (L/min)**	**SPCC (%)**
9.68	0.02	0.36	9.66	0.02	1.20
48.08	0.02	1.78	49.06	0.02	6.11
93.79	0.02	3.51	90.10	0.02	11.23
184.60	0.02	6.90	99.07	0.02	12.35
270.15	0.02	10.10	-	-	-

Supplementary Table 4. Summary of detected substances and corresponding band positions for in situ ATR-IR.

Assignment	Band center (cm^{-1})	Reference
CO_2 (aq) stretching	~2343	*J. Phys. Chem. C* **2019**, *123*, 23898−23906<br>*Sci. Adv.* **2022**, *8*, eabo0399<br>*J. Am. Chem. Soc.* **2017**, *139*, 15664−15667
$*CO_L$ stretching	~1890-1945	*ACS Energy Lett.* **2019**, *4*, 1778−1783<br>*Angew. Chem. Int. Ed.* **2020**, *59*, 12664–12668<br>*Angew. Chem. Int. Ed.* **2021**, *60*, 1–6
C=O (H_2CO_3) stretching	~1740-1780	*Science* **1998**, *279*, 1332–1335.<br>*J. Am. Chem. Soc.* **2013**, *135, 7732–7737*<br>*J. Raman Spectrosc.* **2012**, *43*, 108–115
H−O−H bending	~1580-1650	*ACS Cent. Sci.* **2016**, *2*, 522−528<br>*J. Am. Chem. Soc.* **2020**, *142*, 11750−11762<br>*ACS Energy Lett.* **2019**, *4*, 1778−1783
C=O (*COOH) stretching	~1580-1620	*ACS Catal.* **2017**, *7*, 606−612<br>*J. Am. Chem. Soc.* **2021**, *143*, 18233−18241<br>*J. Phys. Chem. C* **2019**, *123*, 5951−5963
C=O (CO_3^{2-}) stretching	~1490-1500	*J. Phys. Chem. C* **2016**, *120*, 17334−17341<br>*J. Phys. Chem. C* **2019**, *123*, 5951−5963<br>*ACS Catal*. **2015**, *5*, 3148−3156
CO_3^{2-}/HCO_3^- stretching	~1400-1420	*ACS Catal*. **2020**, *10*, 8049−8057<br>*J. Am. Chem. Soc*. **2021**, *143*, 18233−18241<br>*ACS Energy Lett.* **2019**, *4*, 682−689
C−O (*COOH) stretching	~1395-1420	*ACS Catal.* **2017**, *7*, 606−612<br>*Adv. Funct. Mater.* **2021**, 2104243<br>*Angew. Chem. Int. Ed.* **2022**, *61*, e202113918
OH (*COOH) deformation	~1288	*ACS Catal.* **2017**, *7*, 606−612<br>*ACS Energy Lett.* **2019**, *4*, 682−689
HCO_3^- stretching	~1210-1250	*J. Phys. Chem. C* **2019**, *123*, 5951−5963<br>*J. Am. Chem. Soc.* **2020**, *142*, 11750−11762<br>*ACS Nano 2022, 16, 2110−2119*

Reference

[1] Y. Zhang, Z. Zhou, Y. Shen, Q. Zhou, J. Wang, A. Liu, S. Liu, Y. Zhang, *ACS Nano* **2016**, *10*, 9036-9043.

[2] L. Huang, Z. Hu, H. Jin, J. Wu, K. Liu, Z. Xu, J. Wan, H. Zhou, J. Duan, B. Hu, J. Zhou, *Advanced Functional Materials* **2020**, *30*, 1908486.

[3] X. Sun, Y. Tuo, C. Ye, C. Chen, Q. Lu, G. Li, P. Jiang, S. Chen, P. Zhu, M. Ma, J. Zhang, J. H. Bitter, D. Wang, Y. Li, *Angew. Chem. Int. Ed.* **2021**, *60,* 23614-23618.

[4] W. Ren, X. Tan, W. Yang, C. Jia, S. Xu, K. Wang, S. C. Smith, C. Zhao, *Angewandte Chemie International Edition* **2019**, *58*, 6972-6976.

[5] Q. Wang, K. Liu, J. Fu, C. Cai, H. Li, Y. Long, S. Chen, B. Liu, H. Li, W. Li, X. Qiu, N. Zhang, J. Hu, H. Pan, M. Liu, *Angewandte Chemie International Edition* **2021**, *60,* 25242-25245.

[6] Z. Zhou, Y. Shen, Y. Li, A. Liu, S. Liu, Y. Zhang, *ACS Nano* **2015**, *9*, 12480-12487.

[7] B. Yuan, Z. Chu, G. Li, Z. Jiang, T. Hu, Q. Wang, C. Wang, *Journal of Materials Chemistry C* **2014**, *2*, 8212-8215.

[8] H. Yu, R. Shi, Y. Zhao, T. Bian, Y. Zhao, C. Zhou, G. I. N. Waterhouse, L. Z. Wu, C. H. Tung, T. Zhang, *Advanced Materials* **2017**, *29*, 1605148.

[9] V. W. h. Lau, I. Moudrakovski, T. Botari, S. Weinberger, M. B. Mesch, V. Duppel, J. Senker, V. Blum, B. V. Lotsch, *Nature Communications* **2016**, *7*, 12165.

[10] W. Wang, H. Zhang, S. Zhang, Y. Liu, G. Wang, C. Sun, H. Zhao, *Angewandte Chemie International Edition* **2019**, *58*, 16644-16650.

[11] Y. Li, P. Li, J. Wang, Y. Yang, W. Yao, Z. Wei, J. Wu, X. Yan, X. Xu, Y. Liu, Y. Zhu, *Applied Catalysis B: Environmental* **2018**, *225*, 519-529.

[12] J. Song, F. Wang, X. Yang, B. Ning, M. G. Harp, S. H. Culp, S. Hu, P. Huang, L. Nie, J. Chen, X. Chen, *Journal of the American Chemical Society* **2016**, *138*, 7005-7015.

[13] a) C. Jia, X. Tan, Y. Zhao, W. Ren, Y. Li, Z. Su, S. C. Smith, C. Zhao, *Angewandte Chemie International Edition* **2021**, *60,* 23342-23348; b) Y. Li, B. Wei, M. Zhu, J. Chen, Q. Jiang, B. Yang, Y. Hou, L. Lei, Z. Li, R. Zhang, Y. Lu, *Advanced Materials* **2021**, *33*, 2102212; c) Y. Zhang, L. Jiao, W. Yang, C. Xie, H. L. Jiang, *Angewandte Chemie International Edition* **2021**, *60*, 7607-7611; d) E. Zhang, T. Wang, K. Yu, J. Liu, W. Chen, A. Li, H. Rong, R. Lin, S. Ji, X. Zheng, Y. Wang, L. Zheng, C. Chen, D. Wang, J. Zhang, Y. Li, *Journal of the American Chemical Society* **2019**, *141*, 16569-16573; e) Z. Li, D. He, X. Yan, S. Dai, S. Younan, Z. Ke, X. Pan, X. Xiao, H. Wu, J. Gu, *Angewandte Chemie International Edition* **2020**, *n/a*; f) H. L. Jiang, L. Jiao, W. Yang, G. Wan, R. Zhang, X. Zheng, H. Zhou, S. H. Yu, *Angewandte Chemie International Edition* **2020**, *59,* 20589–20595; g) T. Z. X. H. H. Yang, *Angewandte Chemie International Edition* **2020**, *132*, 12153-12159; h) T. Wang, X. Sang, W. Zheng, B. Yang, S. Yao, C. Lei, Z. Li, Q. He, J. Lu, L. Lei, L. Dai, Y. Hou, *Advanced Materials* **2020**, *32*, 2002430; i) J. Pei, T. Wang, R. Sui, X. Zhang, D. Zhou, F. Qin, X. Zhao, Q. Liu, W. Yan, J. Dong, L. Zheng, A. Li, J. Mao, W. Zhu, W. Chen, Z. Zhuang, *Energy & Environmental Science* **2021**, *14*, 3019-3028; j) Z. Zeng, L. Y. Gan, H. Bin Yang, X. Su, J. Gao, W. Liu, H. Matsumoto, J. Gong, J. Zhang, W. Cai, Z. Zhang, Y. Yan, B. Liu, P. Chen, *Nature Communications* **2021**, *12*, 4088; k) B. Zhang, J. Zhang, J. Shi, D. Tan, L. Liu, F. Zhang, C. Lu, Z. Su, X. Tan, X. Cheng, B. Han, L. Zheng, J. Zhang, *Nature Communications* **2019**, *10*, 2980; l) S. Jin, Y. Ni, Z. Hao, K. Zhang, Y. Lu, Z. Yan, Y. Wei, Y.-R. Lu, T. S. Chan, J. Chen, *Angewandte Chemie International Edition* **2020**, *59*, 21885-21889.

[14] W. Ni, Z. Liu, Y. Zhang, C. Ma, H. Deng, S. Zhang, S. Wang, *Advanced Materials* **2020**, *33*,

2003238.
